# Characterisation of lung macrophage subpopulations in COPD patients and controls

**DOI:** 10.1038/s41598-017-07101-2

**Published:** 2017-08-02

**Authors:** Jennifer A. Dewhurst, Simon Lea, Elizabeth Hardaker, Josiah V. Dungwa, Arjun K. Ravi, Dave Singh

**Affiliations:** 1 The University of Manchester; Division of Infection, Immunity and Respiratory Medicine, School of Biological Sciences, Faculty of Biology, Medicine and Health, Manchester Academic Health Science Centre, The University of Manchester and University Hospital of South Manchester, NHS Foundation Trust, Manchester, UK; 2 Novartis Institutes for BioMedical Research, Respiratory Diseases Area, Horsham, UK

## Abstract

Lung macrophage subpopulations have been identified based on size. We investigated characteristics of small and large macrophages in the alveolar spaces and lung interstitium of COPD patients and controls. Alveolar and interstitial cells were isolated from lung resection tissue from 88 patients. Macrophage subpopulation cell-surface expression of immunological markers and phagocytic ability were assessed by flow cytometry. Inflammatory related gene expression was measured. Alveolar and interstitial macrophages had subpopulations of small and large macrophages based on size and granularity. Alveolar macrophages had similar numbers of small and large cells; interstitial macrophages were mainly small. Small macrophages expressed significantly higher cell surface HLA-DR, CD14, CD38 and CD36 and lower CD206 compared to large macrophages. Large alveolar macrophages showed lower marker expression in COPD current compared to ex-smokers. Small interstitial macrophages had the highest pro-inflammatory gene expression levels, while large alveolar macrophages had the lowest. Small alveolar macrophages had the highest phagocytic ability. Small alveolar macrophage CD206 expression was lower in COPD patients compared to smokers. COPD lung macrophages include distinct subpopulations; Small interstitial and small alveolar macrophages with more pro-inflammatory and phagocytic function respectively, and large alveolar macrophages with low pro-inflammatory and phagocytic ability.

## Introduction

Chronic Obstructive Pulmonary Disease (COPD) is characterised by an abnormal inflammatory response to the inhalation of noxious particles such as cigarette smoke^1^. Macrophage numbers are increased in the small airways of COPD patients^2^, and there is a correlation between macrophage numbers and COPD severity^2, 3^. Macrophages provide host defence against pathogens, including bacteria, by phagocytosis and activation of both innate and adaptive immune responses through pathogen recognition receptors and antigen presentation respectively^4, 5^.

Macrophages exhibit a degree of plasticity in response to the extracellular environment^6^, described by the M1/M2 model of macrophage polarisation^7^. M1 macrophages have pro-inflammatory and cytotoxic properties, and are characterised by Human Leukocyte Antigen (HLA)-DR, Cluster of Differentiation (CD) 14 and CD38 expression. M2 macrophages have anti-inflammatory and tissue repair functions, and are characterised by CD36, CD206, and CD163 expression^8–10^. This model is simplistic, as macrophages may have both M1 and M2 characteristics^11, 12^. Nevertheless, it is clear that macrophages may form heterogeneous subpopulations with different physiological functions.

It is known that lung macrophages are capable of replication to maintain cell numbers^13^, and that monocytes can be recruited into the lungs and differentiate into macrophages^14, 15^. Different macrophage subsets have been identified in healthy human lungs, classified as alveolar macrophages, tissue derived monocyte/macrophages and monocyte derived cells^16^. The movement of monocytes and macrophages within the lungs is not well described, but it is possible that these cells may move between different compartments e.g. from the tissue into the airways.

COPD alveolar macrophages differ from healthy controls, releasing lower levels of pro-inflammatory cytokines in response to Lipopolysaccharide (LPS) stimulation^17, 18^, and showing suppression of M1 related genes^19^. COPD alveolar macrophages also have a reduced ability to efferocytose apoptotic epithelial cells^20^ and phagocytose bacteria^21^. COPD macrophages appear to adopt a unique phenotype in response to local environmental conditions of oxidative stress and inflammation. “Small” and “large” macrophage subpopulations have been identified in the sputum of COPD patients^22, 23^, with COPD small macrophages producing increased levels of pro-inflammatory mediators compared to large macrophages^23^. Small macrophages have also been identified within the lower airways of patients with cystic fibrosis and interstitial lung disease^24–26^, while COPD macrophage subpopulations of different densities have been identified in the lower airways^27^.

The characteristics of small and large macrophages in the lower airways of COPD patients has not been studied. Similarly, differences between COPD macrophages in the alveolar spaces and the lung interstitium have not been investigated.

We hypothesized that COPD macrophages in the distal lungs comprise distinct subpopulations that can be defined based on size, and that these subpopulations have different characteristics and functions. This paper reports an investigation of the characteristics of COPD small and large macrophages in the alveolar spaces and lung interstitium.

## Materials and Methods

### Study subjects

**Figure 1:**
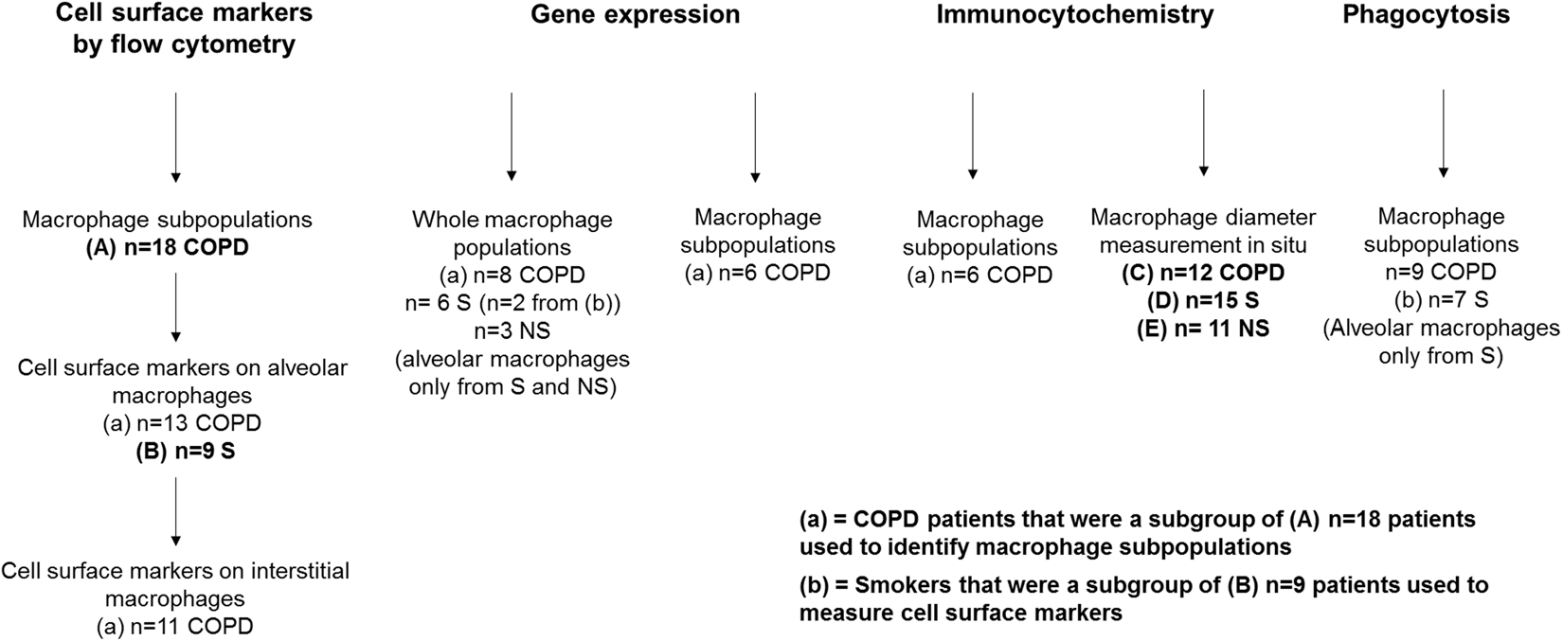
Flow chart describing subsets of patients. a = cells from the same COPD patients used for identifying macrophage subpopulations were used for these experiments, b = cells from the same smoking controls used for measuring cell surface markers on alveolar macrophages were used for these experiments. S: smoking controls, NS: never smokers.

88 patients undergoing surgical resection for suspected lung cancer were recruited and provided cells that were used for experiments as shown in Fig. 1. The demographics of the entire population are shown in Table 1, with the characteristics of patients in each experiment shown in the on-line supplement (Tables S1–S5). COPD was diagnosed based on ≥10 pack years smoking history and GOLD criteria^1^. Controls were smokers without airflow limitation (smoking controls) or never smokers. Ex-smokers were defined as individuals who had stopped smoking for ≥1 year. In all the experiments, COPD patients had lower FEV1% predicted compared to smoking and never smoking controls, and there were no differences in pack year history between COPD patients and smoking controls (Tables S1–S5). COPD current smokers had similar demographic characteristics compared to COPD ex-smokers, with the exception of ex-smokers being older in the flow cytometry experiments. All subjects gave written informed consent. This research was approved by NRES Committee North West- Greater Manchester South (reference 03/SM/396) and all experiments were performed in accordance with relevant guidelines and regulations.

**Table 1:** Subjects’ Demographics.

	COPD	S	NS
n	39	33	16
GOLD stage I	14	N/A	N/A
GOLD stage II	20	N/A	N/A
GOLD stage III	4	N/A	N/A
Age (yrs)	69.5 (6.7)	69.9 (7.8)	75.4 (5.6)
Sex (M/F)	20/19	15/18	4/12
FEV1 (L)	1.6 (0.5)	2.2(0.4)	2.2 (0.5)
FEV1% Predicted	71.2 (14.3)	90.8 (24.3)	114.2 (29.8)
FVC (L)	2.8 (0.7)	2.8 (0.4)	2.8 (0.8)
FEV1/FVC Ratio (%)	56.8 (6.8)	77.4 (23.3)	97.8 (29.6)
Pack Year History	52.6 (6.8)	45.1 (16.6)	0
Current smokers	28	31	N/A
ICS users^#^	14	0	0

Data shown are mean (sd). FEV1: forced expiratory volume, FVC: forced vital capacity, ICS: inhaled corticosteroid. S: Smoking controls, NS: never smokers.

^#^There were no patients on oral steroids.

### Macrophage Isolation

Lung tissue that was far distant and free of tumour was used for experiments, as previously described^28^. Alveolar cells were isolated from resected lung tissue by cannulating the airways and lavaging with 0.1 M NaCl. After lavage the lung tissue was flushed until free of blood before interstitial macrophages were isolated from the lung tissue which was then chopped using a McIlwain™ Tissue Chopper (Campden Instruments, Loughborough, UK) and enzymatically digested. Full methods are described in the online supplement and shown in a flow chart (Fig. S1). Immunohistochemistry of pre- and post-flushed lung tissue for the monocyte/macrophage marker CX_3_CR1 showed both alveolar macrophages (CX_3_CR1 positive cells in alveolar space) and blood monocyte (CX_3_CR1 positive cells in blood vessels) were effectively removed prior to tissue digest for the interstitial population (Fig. S2).

Alveolar and interstitial macrophages were negatively isolated from alveolar and interstitial cells using an EasySep™ human monocyte enrichment kit without CD16 depletion (STEMCELL Technologies, Grenoble, France) according to the manufacturer’s instructions removing cells positive for CD2, CD3, CD19, CD20, CD56, CD66b, CD123 or glycophorin A and red blood cells. Post enrichment granulocyte and lymphocyte content were both <1% in tissue macrophages and <2% and 5% respectively in alveolar macrophages (Fig. S3A and B). Macrophage subpopulations were gated by flow cytometry as shown in Fig. S4. Dendritic cell contamination was ruled out by flow cytometry showing very low levels of cell surface CD123, CD1c or CD1a expression (Fig. S5).

### Measurement of cell- surface markers

Fluorescently labelled antibodies were validated for their sensitivity using monocyte derived macrophages (explanation in online supplement and shown in Table S6). Antibodies chosen for macrophage subpopulation phenotyping were: CD14 (61D3), CD38 (HIT2), HLA-DR (LN3), CD163 (GH1/61), CD206 (19.2) (all eBioscience, Hatfield, UK), and CD36 (CB38) (BD Biosciences, Oxford, United Kingdom). Flow cytometry method details are in the online supplement. Data are expressed as the percentage of positive cells or median fluorescence intensity (MFI).

### Gene expression

Alveolar and interstitial macrophages were sorted into subpopulations using a BD Influx cell sorter (BD Biosciences), and RNA was obtained as described in the on-line supplement. Gene expression was analysed using a Custom Made Taqman Assay for gene panels and Taqman gene expression assay for MARCO (Applied Biosystems) as described in the on-line supplement.

### Measurement of alveolar and tissue macrophage diameter in lung resected tissue

Tissue blocks were obtained from an area of the lung as far distal to the tumour as possible, and processed as described previously^29^. Blocks were dual labelled using antihuman CX_3_CR1 and CD14 primary antibodies. Further details of methods and antibodies are described in the on-line supplement. The number on macrophages were calculated and the diameter of each measured (see on-line supplement).

### Measurement of CXCL1 and TLR3 by Immunocytochemistry

Immunocytochemical analysis of CXCL1 and Toll- like receptor (TLR) 3 from cytospins of alveolar and interstitial macrophages is described in the on-line supplement.

### Measurement of phagocytosis

Phagocytosis of pHrodo™ green Escherichia coli BioParticles (Life Technologies) by alveolar and interstitial macrophages for 1 hour was quantified by flow cytometry as described in the on-line supplement.

### Statistics

All statistical analysis was performed using GraphPadInStat (GraphPad Software Inc, La Jolla, California, USA). All data were normally distributed apart from macrophage diameter data. Paired t tests were used to compare flow cytometry cell surface marker expression and gene expression data between small and large macrophages, and between interstitial and alveolar macrophages. Unpaired t tests were used to compare cell surface marker expression, gene expression and phagocytosis data between patient groups. Mann-whitney ttests were performed to compare cell diameters between alveolar and interstitial macrophages, while One- way ANOVA followed by Tukey- Kramer Multiple Comparisons Test was used to compare cell diameters between subject groups. One- way ANOVA followed by Tukey- Kramer Multiple Comparisons Test was also used to compare phagocytosis and immunocytochemistry data between small alveolar, large alveolar and small interstitial macrophages. For gene expression analysis, a fold change (FC) >1.5 and p < 0.01 was considered significant; this approach uses a lower p value to account to multiple comparisons, while also a FC threshold for biological relevance^30^. P < 0.05 was considered significant in all other analysis.

## Results

### Flow Cytometry

Flow cytometry was performed using cells from 18 COPD patients, of which 13 were used for cell surface marker expression, and 9 smoking controls.

#### Identification of small and large macrophages

**Figure 2:**
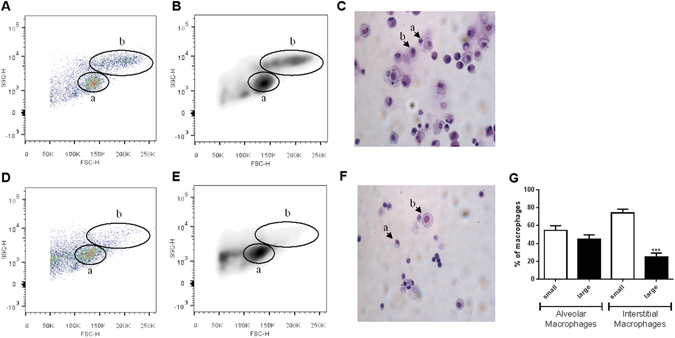
Identifying macrophage subpopulations in alveolar and interstitial macrophages. Alveolar macrophage (**A**,**B** and **C**) and interstitial macrophage (**D**,**E** and **F**) subpopulations were compared and data is shown of enriched macrophages from an ex smoking COPD patient. Flow cytometry pseudocolor plots **(A** and **D** and density plots (**B** and **E**) indicating size (FSC) and granularity (SSC) were used to identify small (a) and large (b) macrophage subpopulations. H&E stained cytospins (**C** and **F**) were used to confirm the presence of small (a) and large (b) macrophages. The number of small and large macrophages present was expressed as a percentage of the total macrophage population (**G**). Data represents mean (SEM) of n = 18 COPD patients. Paired t test (two tailed) was performed. ***The percentage of large interstitial macrophages is significantly decreased compared to the percentage of large alveolar macrophages (p < 0.001).

Isolated macrophages from 18 COPD patients were used to identify subpopulations of small and large macrophages based on size (FSC) and granularity (SSC) (Fig. 2). These subpopulations were also observed microscopically in both alveolar and interstitial macrophages (Fig. 2C and F respectively). Alveolar macrophages consisted of similar numbers of small and large cells (p = 0.11; Fig. 2G), while interstitial macrophages were mainly small rather than large cells (means 74.5% versus 25.5% respectively, p < 0.0001). This pattern was observed in both current and ex-smokers for alveolar macrophages (small 52% versus large 48%; small 54% versus large 46% respectively) and for interstitial macrophages (small 78% versus large 21%; small 70% versus large 29% respectively).

#### Cell surface marker expression in COPD macrophages

Cell surface marker expression on alveolar macrophages was studied in 13 COPD patients, with interstitial macrophages isolated from 11 of these patients using the gating strategy shown in Fig. S4.

**Figure 3:**
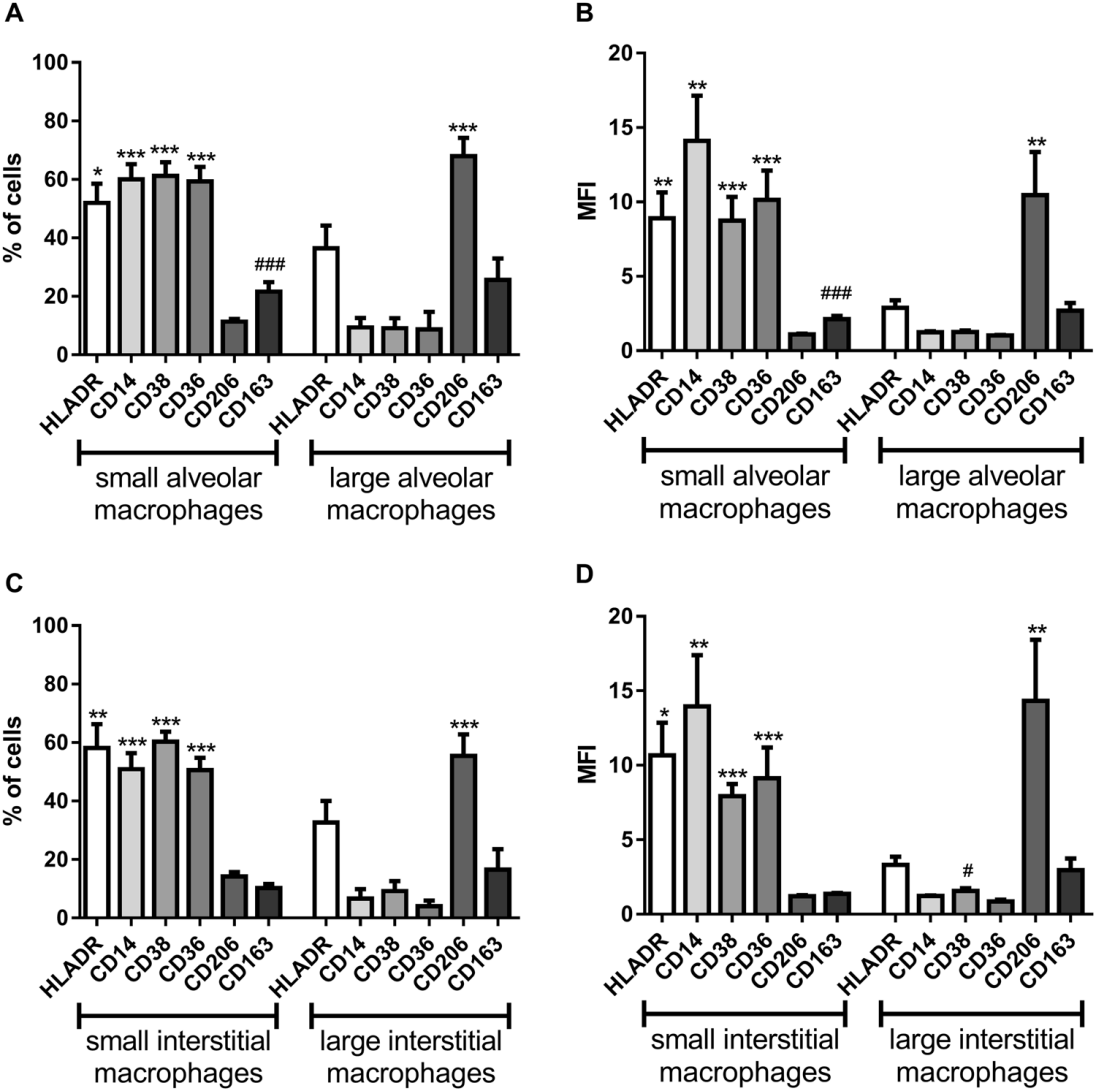
The expression of macrophage markers in small macrophages compared to large macrophages. Flow cytometric analysis of enriched alveolar (**A** and **B**) and interstitial (**C** and **D**) macrophages for n = 13 and n = 11 COPD patients respectively per marker. Markers analysed were HLA-DR, CD14, CD38, CD36, CD206 and CD163. Data is expressed as the percentage of cells within each subpopulation expressing a specific marker (**A** and **C**) and the median fluorescence intensity (MFI) of each marker (**B** and **D**) and represents mean (SEM). Paired t test (two tailed) was performed for each marker. *,**,***Significantly increased between size populations within tissue compartment (p < 0.05, 0.01 and 0.001 respectively). ^#,##,###^Significantly increased between tissue compartments for each size population (p < 0.05, 0.01 and 0.001 respectively)

Small versus large macrophages: In both alveolar and interstitial cells, small macrophages expressed significantly higher HLA-DR, CD14, CD38 and CD36 and lower CD206 compared to large cells (p < 0.05 for each comparison of cell percentages and MFI; Fig. 3). No other cell surface markers showed differences for both cell percentages and MFI on small versus large macrophages.

Alveolar versus interstitial macrophages: CD163 expression (both percentage and MFI) was higher in small alveolar compared to small interstitial macrophages (Fig. 3). No other cell surface markers showed differences for both cell percentages and MFI on small alveolar compared to small interstitial macrophages, or for large alveolar compared to large interstitial macrophages.

**Figure 4:**
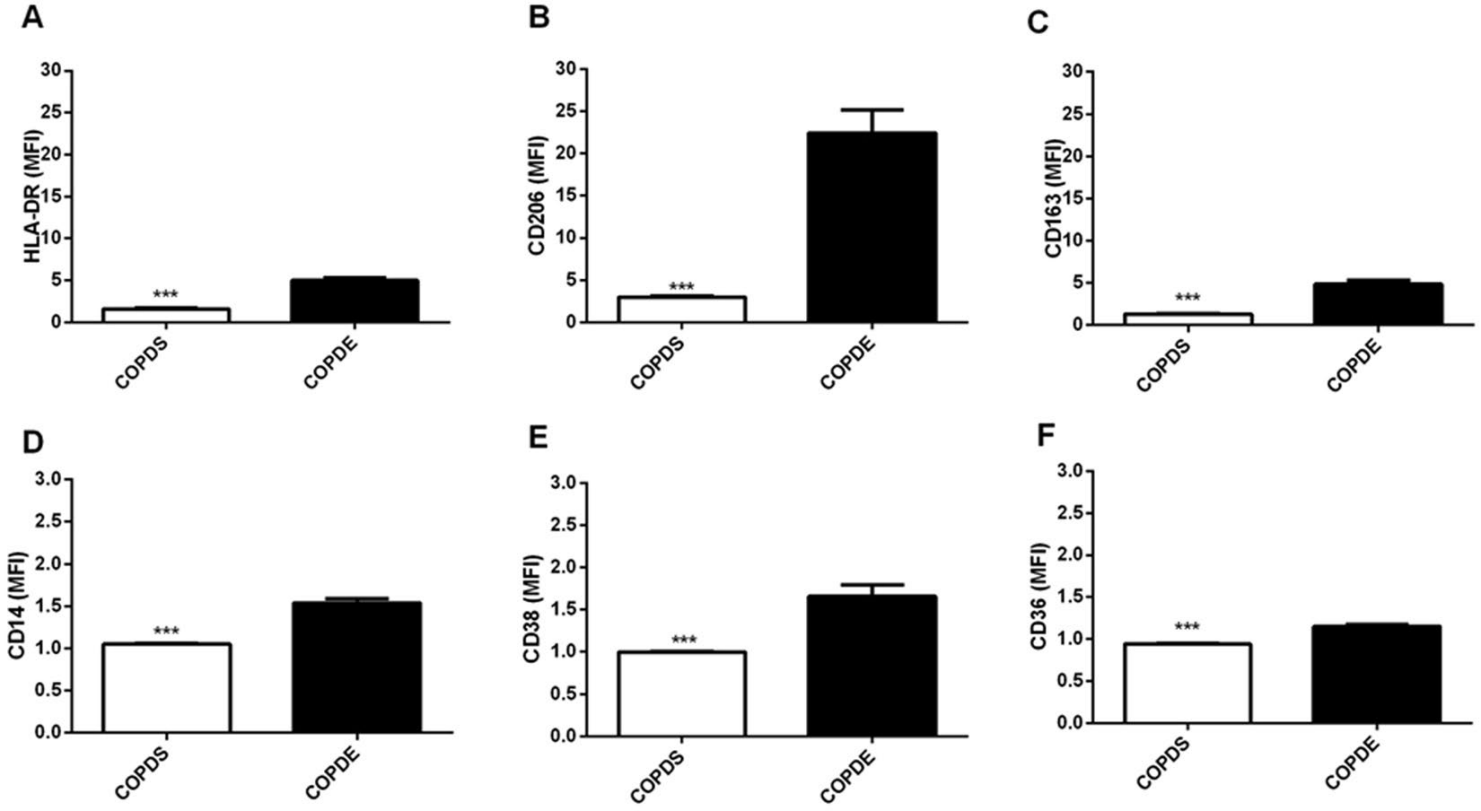
The expression of macrophage markers in the large alveolar macrophage population in COPD (including smoking status). Flow cytometric analysis of large alveolar macrophages for n = 7 COPD smokers (COPDS) and n = 6 COPD ex-smokers (COPDE). Markers analysed were HLA-DR (**A**), CD206 (**B**), CD163 (**C**), CD14 (**D**), CD38 (**E**) and CD36 (**F**). Data is expressed as the median fluorescence intensity (MFI) of each marker and represents mean (SEM). Unpaired t test (Two-tailed) was performed for each marker. ***Significantly different compared to expression of the same marker on large alveolar macrophages from COPDE patients (p < 0.001).

Current versus ex-smoking COPD patients: COPD alveolar macrophages were from 7 current and 6 ex-smokers. Large alveolar macrophages showed lower MFI of all markers in current smokers (Fig. 4), with a similar pattern for cell percentages (Table S7). For small alveolar macrophages only HLA-DR showed a decrease in current smokers (Table S7).

Interstitial macrophages were from 6 current smokers and 5 ex-smokers. For large interstitial macrophages, there were significant decreases in CD38 and CD163 MFI caused by current smoking, with numerical trends for a decrease in these markers for cell percentages (Table S7). For small interstitial macrophages, there were few differences between groups with no consistent patterns observed for both MFI and cell percentage results (Table S7).

Sub-analysis of COPD patients using and not using ICS compared (3 versus 10 patients respectively) showed no difference between groups for expression of cell surface markers (Fig. S7).

#### Surface markers on COPD patients versus smoking controls

Cell surface marker expression in COPD alveolar macrophages was compared to smoking controls. The proportions of small and large alveolar macrophages in 18 COPD patients (52% and 48% respectively) and 9 smoking controls (59% and 40% respectively) were similar. CD206 expression on small alveolar macrophages was significantly decreased in COPD patients (n = 13) compared to smoking controls (n = 9; 11% versus 26% respectively, p = 0.002; p = 0.02 for MFI analysis) (Table S8). No other differences between subject groups were observed for both MFI and cell percentage results.

### Gene expression analysis

#### Alveolar versus interstitial macrophages in COPD patients

Gene expression in alveolar and interstitial macrophages was compared in 8 COPD patients. 67 out of 179 genes measured were significantly changed (Table S9); the majority had increased expression levels in interstitial macrophages, with the most highly regulated shown in Table 2. Alveolar macrophages from 5 COPD current smokers were compared to 3 COPD ex-smokers. There were 6 out of 179 genes with FC > 1.5 (p < 0.01); these are listed in Table S10. No gene expression changes (FC > 1.5;p < 0.01) were observed in interstitial macrophages from COPD current smokers compared to ex-smokers.

**Table 2:** Genes showing significantly different expression in whole interstitial, small interstitial and large alveolar macrophages from COPD patients.

Gene name	Gene ID	*P* Value	Fold change	Function
**Interstitial macrophages v Alveolar macrophages**				
NLRP3	Hs00918082_m1	0.0009	20.08	Inflammation /// immune response /// apoptosis
IL1B	Hs01555410_m1	0.0039	16.19	Cytokine /// inflammation /// cell proliferation and differentiation /// apoptosis
IL1RL1	Hs00545033_m1	0.0004	15.76	Cytokine receptor /// pro-inflammatory
IL6	Hs00985639_m1	0.0016	15.48	Cytokine /// pro and anti-inflammatory
SFTPD	Hs00358340_m1	0.0002	13.62	Surfactant protein /// host defence
IL10	Hs00961622_m1	0.0002	13.59	Cytokine /// immunoregulatory
MUC5B	Hs00861595_m1	0.0002	12.97	Mucin /// host defence
SFTPA1	Hs00831305_s1	0.0005	12.8	Surfactant protein /// host defence
CXCL1	Hs00605382_g1	0.0001	12.19	Growth factor /// neutrophil chemoattractant
MUC1	Hs00159357_m1	0.0001	12.11	Mucin /// host defence
**Small Interstitial macrophages v Small Alveolar macrophages**				
CCL20	Hs01011368_m1	0.0050	114.21	Lymphocyte chemoattractant
CXCL1	Hs00605382_gH	0.0018	16.56	Growth factor /// neutrophil chemoattractant
LTF	Hs00914334_m1	0.0026	11.91	Transferrin /// iron homeostasis /// host defence /// anti-inflammatory
INHBB	Hs00173582_m1	0.0006	11.06	Inhibin /// negative regulator of cell proliferation /// tumour-suppressor
CCL3	Hs00234142_m1	0.0073	10.63	Acute inflammation /// granulocyte recruitment
MUC5B	Hs00861595_m1	0.0011	10.09	Mucin /// host defence
ICAM1	Hs00164932_m1	0.0063	9.77	Adhesion /// transmigration
VEGFA	Hs00900055_m1	0.0066	6.26	Growth factor /// angiogenesis /// cell migration /// apoptosis inhibition
IL1R1	Hs00991002_m1	0.0061	5.44	Cytokine receptor /// pro-inflammatory
TLR3	Hs01551078_m1	0.0093	5.36	Host defense against viruses
**Large Alveolar macrophages v Small Alveolar macrophages**				
INHBA	Hs01081598_m1	0.0005	31.31	Inhibin and activin /// negative regulation of cell proliferation
CXCL5	Hs00171085_m1	0.0030	24.42	Neutrophil chemotaxis
VEGFA	Hs00900055_m1	0.0006	−6.67	Growth factor /// angiogenesis /// cell migration /// apoptosis inhibition
SFTPD	Hs00358340_m1	0.0042	−7.14	Surfactant protein /// host defence
CCL17	Hs00171074_m1	0.0007	−7.14	T cell chemotaxis
TLR2	Hs01872448_s1	<0.0001	−7.69	Host response to Gram-positive bacteria and yeast
NLRP3	Hs00918082_m1	0.0004	−9.09	Inflammation /// immune response /// apoptosis
IL1R2	Hs01030384_m1	0.0007	−10.00	Decoy receptor /// inhibitory signal /// anti-inflammatory
SFTPA1	Hs00831305_s1	0.0006	−14.29	Surfactant protein /// host defence
CCL22	Hs01574247_m1	<0.0001	−16.67	Chemokine /// trafficking of activated T lymphocytes to inflammatory sites

The 10 most highly up and down regulated genes are shown. The full lists of genes are shown in Tables S9, S11 and S12. Macrophage mRNA was analysed using a Custom Made Taqman Assay and data was analysed with whole alveolar macrophages or small alveolar macrophages as the reference. Paired t test (two tailed) was performed and p < 0.01 for all genes.

#### Subpopulations of alveolar versus interstitial macrophages in COPD patients

Alveolar and interstitial macrophages were obtained from a different group of 6 COPD patients (demographics in Table S2) and isolated into large and small subpopulations; there were lower numbers of large interstitial cells resulting in insufficient RNA for analysis.

17 genes had significantly higher expression in small interstitial compared to small alveolar macrophages (Table S11). 15 of these genes were also different in whole interstitial compared to whole alveolar macrophages.

42 genes were significantly different in large alveolar compared to small alveolar macrophages (Table S12). The majority of these genes had lower expression in large alveolar macrophages, including IL-10 and vascular endothelial growth factor (VEGF) with fold changes of 0.29 (p = 0.002) and 0.15 (p = 0.0006) respectively.

The 10 most highly up and down regulated genes in whole interstitial, small interstitial and large alveolar macrophages are shown in Table 2. Four genes (interleukin 1 receptor (IL1R) 1, intracellular adhesion molecule 1 (ICAM1), VEGFA and IL1R-like 1 (IL1RL1)) were significantly changed in all 3 analysis; all were increased in interstitial compared to alveolar macrophages (both the whole population and small macrophages only), and were decreased in large compared to small alveolar macrophages.

RNA was isolated from the macrophage subpopulations from a further 8 COPD patients (demographics in Table S2) for the analysis of MARCO expression. MARCO expression was significantly increased in large alveolar macrophages (p < 0.001) compared to both small alveolar and small interstitial macrophages (Fig. S8).

#### COPD patients versus smoking and never smoker controls

Gene expression changes in the whole alveolar macrophage population were compared using cells from 8 COPD patients, 6 smoking controls and 3 never smokers. There were 5 genes out of 179 significantly changed in COPD patients compared to smoking controls (Table S13). There were 29 genes significantly changed in COPD patients compared to never smokers, with the majority of genes (n = 24) showing up regulation in COPD patients (Table S14). Upregulated genes included those involved in viral recognition or anti-viral response (e.g. toll-like receptor 7 and interferon α receptor) and apoptosis genes, while down-regulated genes included the anti-oxidant superoxide dismutase 1. Only 1 gene was significantly changed in smoking controls compared to never smokers.

### Immunocytochemistry

#### Cell diameter of macrophage subpopulations

**Figure 5:**
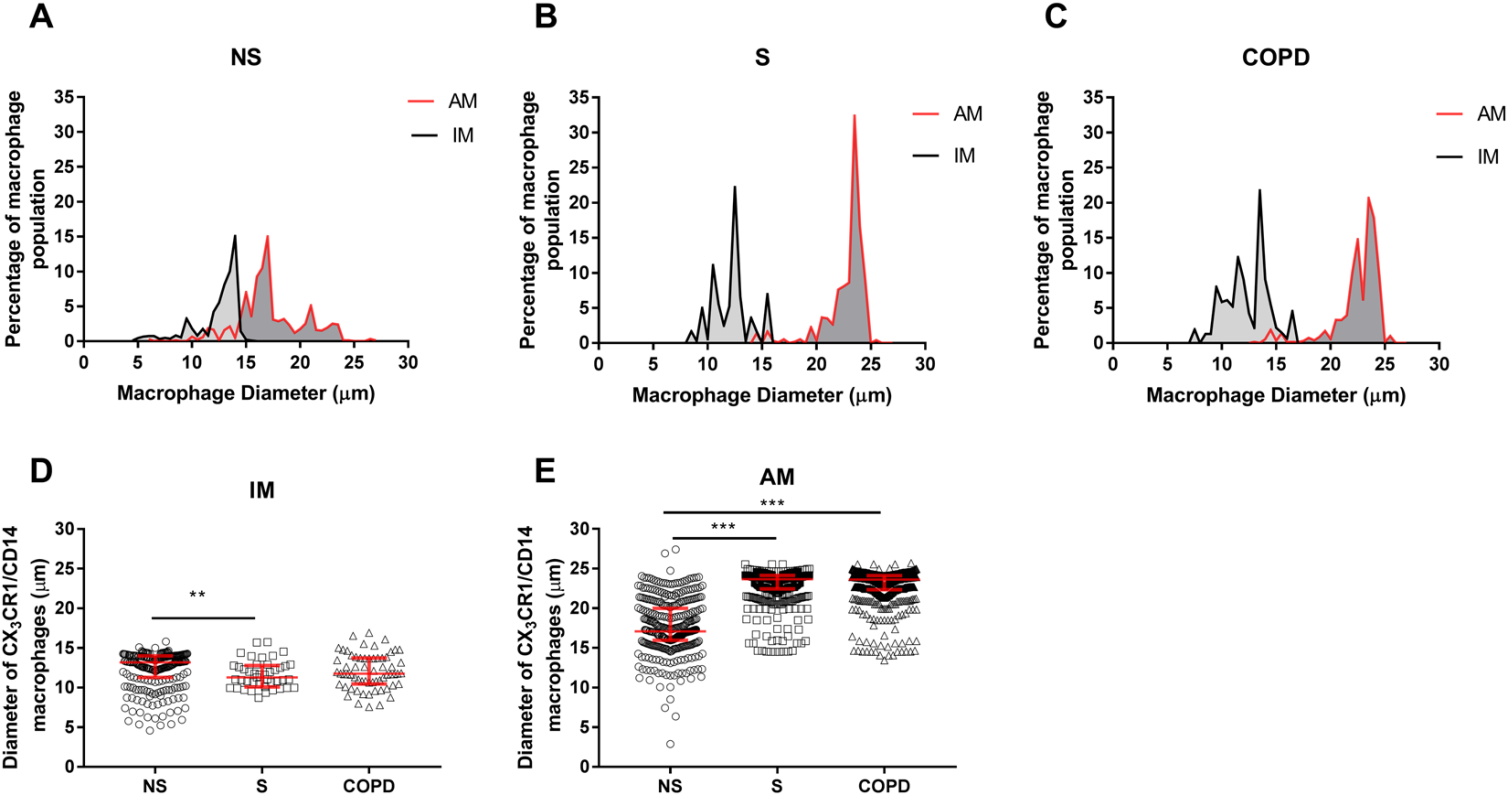
The diameter of alveolar and interstitial macrophages in lung resected tissue. Alveolar (AM) and interstitial (IM) macrophages from never smokers (NS) (n = 11) (**A**), smoking controls (S) (n = 15) (**B**) and COPD patients (COPD) (n = 12) (**C**) were identified in FFPE resected lung tissue by co-expression of CX_3_CR1 and CD14 and the diameter of each cell was measured. Data are shown as the percentage of macrophage population measuring a certain diameter (**A**-**C**) or individual cell diameters for IMs (**D**) and AMs (**E**) with median and interquartile range. Mann-whitney t-tests were performed between IM and AMs. One way ANOVA followed by Tukey-Kramer Multiple Comparisons Test were performed between subject groups. **,***Significant difference (p < 0.01, < 0.001 respectively)

Macrophages were identified in peripheral lung tissue from 12 COPD patients, 15 smoking controls and 11 never smokers by expression of CX_3_CR1 and CD14; all CX_3_CR1 +ve cells also showed co-expression of CD14. Macrophages located in the alveolar spaces were defined as alveolar macrophages (AM) (white and red arrows Fig. S9) and macrophages located in the alveolar walls or peripheral tissue were defined as interstitial macrophages (IM) (yellow arrows Fig. S9). The median diameter of the alveolar macrophage population was significantly larger than the interstitial macrophage population for never smokers (17.1 µm and 13.2 µm respectively), smoking controls (23.7 µm and 11.3 µm respectively) and COPD patients (23.7 µm and 11.8 µm respectively) (p < 0.001 for all comparisons) Fig. 5A–C. The median diameter of alveolar macrophages was significantly greater in both smoking controls and COPD patients compared to never smokers (p < 0.001 for both comparisons) (Fig. 5E). Interstitial macrophage diameter was significantly greater in never smokers compared to smoking controls (p < 0.01) (Fig. 5D).

Carbon deposits were observed in the cytoplasm of larger cells using light microscopy (Fig. S9D black arrows), although large macrophages were also observed without carbon deposits (Fig. S9D green arrows).

#### CXCL1 and TLR3 expression in isolated macrophage populations

**Figure 6:**
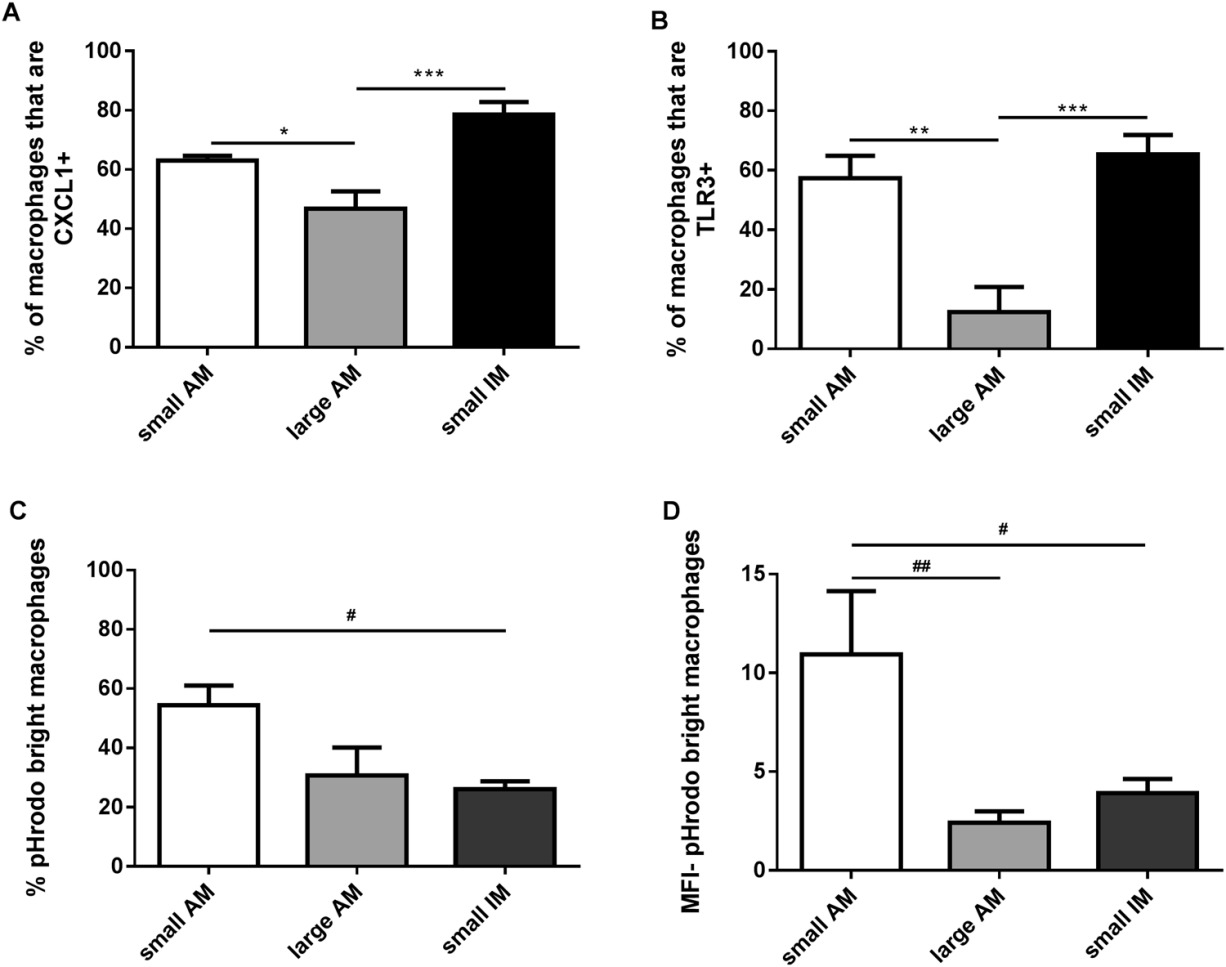
Expression of CXCL1 and TLR3 protein and phagocytosis of pHrodo E. coli BioParticles in macrophage subpopulations. Alveolar (AM) and interstitial (IM) macrophages were isolated using EasySep monocyte enrichment kit. Cells were used to generate cytospin slides (**A** and **B**) or exposed to pHrodo E. coli BioParticles for 1 h in a shaking incubator (**C** and **D**). Percentage of small alveolar, large alveolar and small interstitial macrophages expressing CXCL1 protein and TLR3 protein are shown in (**A** and **B**) respectively. Data represents mean (SEM) of n = 6 COPD patients. Phagocytosis was analysed by flow cytometry and is represented by (**C**): % of pHrodo bright macrophages within each subpopulation and (**D**): median fluorescence intensity (MFI) of each subpopulation relative to the negative control. Data are shown as mean (SEM) of n = 9 COPD patients One way ANOVA followed by Tukey-Kramer Multiple Comparisons Test was performed. *,**,***Significantly different compared to expression of the same protein on large alveolar macrophages (p < 0.05, < 0.01 and <0.001 respectively). ^#,##^Significantly different compared to the percentage of pHrodo bright macrophages or MFI of other macrophage subpopulations (p < 0.05, <0.01 respectively).

CXCL1 and TLR3 gene expression levels were higher in small interstitial macrophages compared to small alveolar macrophages (Table 2). We sought to validate these findings by immunohistochemistry. CXCL1 and TLR3 protein expression in samples from 6 COPD patients was investigated in the 3 macrophage subpopulations used for gene expression (small and large alveolar, and small interstitial macrophages). CXCL1 and TLR3 protein expression were numerically highest in small interstitial macrophages and lowest in large alveolar macrophages, thus matching the pattern seen for gene expression (Fig. 6A,B and representative images shown in Fig. S10).

### Phagocytosis in COPD alveolar versus interstitial macrophages

Alveolar and interstitial macrophages from 9 COPD patients were used to investigate phagocytosis of E. coli BioParticles. Small alveolar macrophage phagocytosis was significantly higher compared to other macrophage subpopulations, using both the percentage of cells carrying out phagocytosis and MFI (Fig. 6C,D). COPD current (n = 6) and ex-smokers (n = 3) showed no differences for small macrophages, but lower large alveolar phagocytosis in current smokers (Fig. S11). Alveolar macrophages obtained from 6 smoking controls showed greater phagocytosis in small compared to large cells, with similar levels compared to COPD patients (Fig. S11).

## Discussion

We identified four macrophage subpopulations in the lower airways and lung tissue of COPD patients using flow cytometry. Based on size and location, we called these small alveolar, large alveolar, small interstitial and large interstitial macrophages, in line with nomenclature previously used to describe lung macrophages^22, 23^. Small macrophages expressed a distinct profile of cell surface markers compared to large macrophages. Furthermore, small interstitial macrophages had the highest expression of pro-inflammatory genes while large alveolar macrophages had the lowest. Small alveolar macrophages had the greatest phagocytic capacity.

We used immunohistochemistry quantification of cell size to validate our flow cytometry findings showing different macrophage subpopulations. Immunohistochemistry demonstrated a large variation in macrophage cell sizes, confirming the presence of relatively smaller and larger cells. Furthermore, we confirmed our flow cytometry observation that interstitial cells were predominantly smaller cells. Flow cytometry used both size and granularity to distinguish cells, while immunohistochemistry relied on size only. These differences between techniques caution against over-interpretation of the comparison of the proportions of cells categorised as small or large by each method. Nevertheless, both methods showed that larger and smaller cells exist, and that there are proportionally more smaller cells in the interstitium. Additionally, immunohistochemistry provided a comparison against never smokers; smoking controls and COPD patients showed an alveolar macrophage shift towards larger cell size.

Overall, we report a complexity of COPD macrophage phenotypes based on size and location that do not easily fit into the traditional macrophage polarization terminology. It has been accepted for many years that classically activated M1 cells are induced by interferon-γ or LPS and alternatively activated M2 cells are induced by IL-4 or IL-13. More recently, further subtypes of M2 macrophages have been proposed; M2a, M2b and M2c with the idea of macrophage activation existing as a spectrum not a dichotomy^31, 32^. Supporting this concept, Chana *et al*. reported that COPD macrophages have both M1 and M2b properties^27^. The subpopulations identified here add further to the complexity of macrophage phenotyping, and are now discussed.

### Small Macrophages

Small macrophages were characterised as HLA-DR^high^, CD14^high^, CD38^high^, CD36^high^ and CD206^low^ relative to large macrophages. HLA-DR and CD14 are centrally involved the innate immune response to pathogens^33–35^. CD38 controls intracellular calcium levels, thereby enhancing macrophage functions including chemotaxis, cell adhesion, cytokine secretion and phagocytosis^36^. CD36 regulates the efferocytosis of apoptotic neutrophils^37^. This combination of HLA-DR^high^, CD14^high^, CD38^high^ and CD36^high^ therefore seems to identify a pro-inflammatory macrophage subpopulation primed towards enhanced pathogen recognition and phagocytosis. Gene expression analysis supported this concept, as small alveolar macrophages had higher pro-inflammatory gene expression compared to large alveolar macrophages.

Small interstitial macrophages had higher pro-inflammatory gene expression compared to small alveolar macrophages. In contrast, small alveolar macrophages had a greater phagocytic ability compared to small interstitial macrophages. Lung location appears to influence the function of small macrophages.

Different subsets of the mononuclear phagocyte population have been previously categorised in healthy human lungs as alveolar macrophages, tissue derived monocyte/macrophages and monocyte derived cells based on cell surface markers and morphology^16^. These monocyte derived cells are postulated to originate from the blood, and could correspond to our small macrophage populations. This is supported by our finding of low MARCO gene expression in small macrophages; MARCO is expressed at higher levels in embryonic derived alveolar macrophages compared to recruited myeloid cells/blood monocytes^38^. However, we have recently shown lower blood monocyte recruitment into the lungs of COPD patients compared to controls^14^, suggesting that the increased macrophage numbers in COPD derive from alternative mechanisms such as reduced apoptosis^14, 39^.

CD163 expression was significantly higher in small alveolar compared to small interstitial macrophages. CD163 is a scavenger receptor, and CD163+ macrophages play a role in the resolution of inflammation^40^. CD163+ alveolar macrophage numbers increase with COPD severity^41^; these CD163+ alveolar macrophages in severe COPD may be predominantly small cells acting to restrict inflammation.

### Large Macrophages

Large macrophages were characterised as HLA-DR^low^, CD14^low^, CD38^low^, CD36^low^ and CD206^high^ relative to small macrophages. CD206 is involved in pathogen recognition^41^. HLA-DR is involved in antigen-presentation to CD4+ T-cells, and our findings suggest reduced antigen-presentation capacity of large alveolar macrophages. HLA-DR expression is also related to the activation state of alveolar macrophages^42^. One could classify these large alveolar macrophages with low functional capacity as M2c macrophages, which are deactivated macrophages^43^. These cells may be involved in immunoregulation, with low functional capacity to prevent further amplification of inflammation in COPD. However, the reduction in IL-10 and VEGF expression does not match with an “M2” classification, although of course this is a simplistic classification.

Acute cigarette smoke exposure *in vitro* alters macrophage gene expression^44^. Current smoking in COPD patients affected the cell surface marker profile of large macrophages, and not small macrophages; this was most apparent in large alveolar macrophages. The magnitude of changes caused by current smoking were relatively small, and may not be of biological significance. However, the CD206 expression change was relatively large (MFI reduced from 22 to 3) and more likely to be of biological significance. This CD206 down-regulation is compatible with our observation that current smoking decreased COPD large alveolar macrophage phagocytosis capacity.

The higher MARCO gene expression levels in large alveolar macrophages suggest that these cells are derived from embryonic precursors within the lungs^38^ rather than recruited from the blood. We observed carbon deposits in larger macrophages, but the absence of carbon deposits in many large macrophages indicates that the size of these cells is not simply due to carbon deposition associated with cigarette smoking.

### COPD compared to controls

Lung cancer surgery is usually performed on patients with a history of smoking. Never smokers are a minority^45^, so only a few samples from such patients were available. This limited the scope of the flow cytometry study, with never smokers being included only for gene expression and immunohistochemistry studies where historically collected samples were available. However, smokers without airflow obstruction allowed changes due to COPD rather than chronic smoking to be assessed.

CD206 expression was reduced on COPD compared to smokers small alveolar macrophages. Chana *et al*. also reported reduced CD206 expression in COPD macrophages compared to controls^27^. CD206 plays a role in bacterial recognition^41^; and so reduced expression may lead to reduced phagocytosis. Phrodo E. coli BioParticles were used for phagocytosis experiments as a practical and reliable assay. However, E. coli is not commonly isolated in COPD sputum^46^. The lack of difference between COPD patients and controls may be due to the type of bacteria used, as impaired COPD macrophage phagocytosis is restricted to certain bacteria including H. influenza^47^.

Immunocytochemistry analysis of cell diameter showed a clear increase in the size of alveolar macrophages in both smokers and COPD patients compared to never smokers. The differences between groups for interstitial macrophages were small and of doubtful relevance. This shift in size caused by long term smoking in alveolar but not interstitial macrophages further underlines that macrophage phenotype changes can be dependent on the specific pulmonary location.

There were significant gene expression differences between COPD and never smoker alveolar macrophages (29 genes), with fewer differences compared to smoking controls. Nevertheless, these findings confirm previous observations that the phenotype of COPD alveolar macrophages is changed compared to controls^28, 48–51^. Indeed, the results for COPD patients versus never smoking controls included upregulation of genes involved in apoptosis which has been previously reported in COPD^52^.

### Gene expression

The gene expression results were filtered to remove false positive results by using a reduced p value (p < 0.01) and a FC threshold of 1.5. The latter allowed removal of genes that are less likely to be biologically relevant due to a smaller magnitude of change. This approach has been used in gene array studies that analyse >40,000 genes^30, 53^, which will generate more false positives than our analysis of 179 genes. An alternative statistical method to reduce false positive results in gene array studies is false discovery rate (FDR) analysis^54^. Whichever statistical method is used, it is important to further validate the results.

17 genes were significantly changed in small interstitial compared to small alveolar macrophages. In a different group of patients, 15 of these genes were also significantly different in whole interstitial compared to whole alveolar macrophages. These similar results in different patient groups provide a degree of validation. Immunocytochemistry validation was performed for CXCL1 and TLR3 because of the relatively large gene expression differences, and the availability of appropriate immunocytochemistry antibodies. We confirmed that the expression of these proteins was highest in small interstitial macrophages, and lowest in large alveolar macrophages.

The expression of four genes (IL1RL1, ICAM1, VEGFA and IL1R1) was highest in small interstitial macrophages, and lowest in large alveolar macrophages. The function of these genes supports the argument that small interstitial macrophages have the most pro-inflammatory function of the macrophage subsets investigated; IL1R1 is responsible for mediating the pro-inflammatory effects of the IL-1 cytokine family, including IL1RL1^55^. VEGFA has a number of functions relevant to angiogenesis as well as being chemotactic for macrophages^56^ and granulocytes^57^, while ICAM1 is an integrin that can guide chemotaxis in tissues^58^.

The flow cytometry findings combined with the gene expression data indicate that COPD small interstitial macrophages have specialised pro-inflammatory and antigen recognition characteristics, small alveolar macrophages provide phagocytic defence and large alveolar macrophages display a down-regulation of these functions.

### Study limitations

We used lung samples from patients with a diagnosis of cancer. This is a practical approach used in many previous publications^5, 14, 18, 28, 29, 41, 59^. The tissue used was far distant from tumour, reducing any possible influence of the presence of cancer on our results. Furthermore, the similarity of alveolar and interstitial flow cytometry marker expression argues against tumour associated macrophages with unique characteristics being present in our tissue samples, as we would then expect to see a difference in cellular characteristics compared to alveolar cells not in contact with tumour. Overall, it is unlikely that tumour associated macrophages influenced our results. Additionally, corticosteroids can influence the expression of some macrophage markers such as CD163^60^. We did not observe any corticosteroid effects in the flow cytometry analysis, and only a minority of the COPD patients were taking these drugs.

## Conclusions

Distinct subpopulations of macrophages exist in the lower airways of COPD patients and controls. We have identified small interstitial macrophages that are pro-inflammatory, small alveolar macrophages that are highly phagocytic and large alveolar macrophages that have low pro-inflammatory and phagocytic ability. The pharmacological targeting of COPD macrophages could be tailored towards the characteristics of specific subpopulations.

## Electronic supplementary material

Online Supplement
