# Supplementary material for: Characterisation of lung macrophage subpopulations in COPD patients and controls
Source: Sci Rep. 2017 Aug 2;7:7143. doi: 10.1038/s41598-017-07101-2 (PMC5540919; doi:10.1038/s41598-017-07101-2)
Supplement: Supplementary file 1 — Online Supplement [file 41598_2017_7101_MOESM1_ESM.doc]

**Characterisation of lung macrophage subpopulations in COPD patients and controls**

Jennifer A Dewhurst, Simon Lea, Elizabeth Hardaker, Josiah V Dungwa, Arjun K Ravi, Dave Singh

**Supplementary material**

**METHODS**

**Macrophage Isolation**

Alveolar macrophages

Airways of the resected lung tissue were cannulated and lavaged with 0.1 M NaCl. The retrieved fluid was centrifuged (10 min, 400 g, room temperature). The cell pellet was re-suspended in RPMI-1640 media (Sigma-Aldrich, Poole, UK), layered over Ficoll-Paque (GE Healthcare, Buckinghamshire, UK), and centrifuged.

Interstitial macrophages

10g of the post lavage lung tissue was chopped using a McIlwain™ Tissue Chopper (Campden Instruments, Loughborough, UK). Chopped lung tissue was digested with Hanks Balanced Salt Solution (Sigma-Aldrich) supplemented with 0.1% Collagenase (Sigma-Aldrich) and 0.05% DNase I (Sigma-Aldrich) in a water bath (30 min, 37°C). Collagenase and DNase have previously been shown to perform better regarding cell yield and surface markers expression when compared to enzymes that contain neutral proteases, whilst maintaining integrity of cell function and cell surface markers to allow accurate downstream analysis . Cell suspensions were filtered with 100 µm filter, followed by 40 µm filter (Becton Dickenson, Oxford). To maximise cell retrieval interstitial cells did not undergo Ficoll-Paque separation prior to the negative isolation outlined below.

Alveolar and interstitial macrophages were then negatively isolated from cell suspensions using an EasySep™ human monocyte enrichment kit without CD16 depletion (STEMCELL Technologies, Grenoble, France) according to the manufacturer’s instructions removing cells positive for CD2, CD3, CD19, CD20, CD56, CD66b, CD123 or glycophorin A and red blood cells by dextran. Cell number was assessed using Methyl Violet in acetic acid (Neubauer haemocytometer). Above methods are also represented in the flow chart Figure S1.

**Gating strategy for studying macrophage subpopulations**

Alveolar and interstitial macrophages were isolated following EasySep purification. A dot plot was used to identify macrophages based on size (FSC) and granularity (SSC). Events less than 100K FSC-H are likely to be debris and were excluded from analysis by drawing a gate around events greater than 100K FSC-H (P1 Figure S4 upper panel). Only events within this gate (P1) were analysed further as macrophage subpopulations (P2 and P3 Figure S4 bottom panel) for either marker expression or proportions of subpopulations.

**Dendritic cell contamination**

Alveolar and interstitial macrophages were isolated following EasySep purification from 7 control patients (5 smoking controls and 2 never smokers). Cell were analysed for cell surface expression of CD123, CD1c and CD1a. There was minimal CD123 staining in all four macrophage subpopulations (Figure S5). There was also very little staining for the myeloid DC markers CD1c or CD1a in any of the macrophage subpopulations. Masten et al (2006) show myeloid DCs in human lung tissue are characterised as CD1c^+^CD11c^+^CD14^-^HLA-DR^+^ and that plasmocytoid DCs are CD123^+^CD11c^-^CD14^-^HLA-DR^-^ . Here we show neither population to be present in any of the four macrophage subpopulations.

**Measurement of cell- surface markers**

All flow cytometry was carried out using 0.5x10^6^ cells per tube. Cells were incubated with 1 µl per tube of Fc block (eBioscience) at 4°C for 10 min to inhibit non-specific binding of antibodies to the Fc receptors on the surface of cells. Cells were incubated with 1 µl of allophycocyanin (APC) conjugated antibody or the equivalent isotype at 4°C for 30 min. Following washing in PBS (270 *g*, 7 min, room temperature) cells were re-suspended in 400 µl PBS and analysed using BD Canto II with Diva 6.0 software (BD Biosciences). Using FlowJo Vx (FlowJo LLC, Oregon, US) marker expression was quantified against the equivalent isotype control. Due to the autofluorescence of macrophages from COPD patients having been shown to be less in the red channel each cell surface marker was stained for separately using only APC channel . MFI was calculated based on the MFI of cells stained with antibody divided by the MFI of the respective control.

**Isolation of Monocyte Derived Macrophages**

Monocytes were isolated from peripheral blood of never smokers (n=3) and re-suspended at 2x10^6^ml^-1^ in supplemented RPMI 1640 media. On Day 1, monocytes were seeded at 2x10^6^ per well in 6 well plates, stimulated with either 2 ng/µl GM-CSF or 20 ng/µl M-CSF supplemented media and incubated in a 5% CO_2_ humidified atmosphere at 37°C. On Day 3, media was removed and supplemented RPMI 1640 media was added. On Day 5, cells were stimulated with either 2 ng/µl GM-CSF or 20 ng/µl M-CSF supplemented media then some M-CSF differentiated macrophages were further stimulated with 20 ng/µl IL-4 or IFN. On Day 7, media was removed and supplemented RPMI 1640 media was added. MDMs were fully differentiated and ready to use at 12 days. Accutase® solution (Sigma-Aldrich) was added to each well (100 µl) and returned to the incubator for 10 min. Using a Pasteur fine tip pipette, cells were gently scraped and collected in 5 ml tubes, washed by centrifugation in 2 ml PBS, re-suspended in 400 µl PBS for flow cytometry protein experiments. Cells were single stained with antibody and the % positive cells were recorded by flow cytometry.

Antibodies were chosen for macrophage phenotyping studies based on relative sensitivity and specificity for M1 or M2 macrophages (Table S6). Expected markers expressed on M1 differentiated macrophages were CD14, CD38 and HLA-DR. Expected markers expressed on M2 differentiated macrophages were CD163, CD206 and CD36. M1-like MDMs were monocytes differentiated in the presence of GM-CSF alone or M-CSF and IFN. M2-like MDMs were monocytes differentiated in the presence of M-CSF alone or M-CSF and IL-4.

Clones were chosen based on relative levels of expression on M1: M2 differentiated cells and are highlighted in grey in Table S6.

**Measurement of gene expression**

Whole and subpopulations of alveolar and interstitial macrophage mRNA were analysed using a Custom Made Taqman Assay in 384-well Microfluidic cards according to the manufacturer’s instructions (Applied Biosystems: part number 4342265, lot number A8157). cDNA was reacted with TaqMan Universal PCR Master Mix (2x) (Applied Biosystems) according to manufacturer’s instructions. Sample mix was loaded in each port of the microfluidic card and sample was equally distributed throughout the wells by centrifugation (400 *g*, 1 min, room temperature). The microfluidic cards contained premade ABI Taqman gene expression assays. The endogenous controls were GAPDH, ribosomal RNA (18S), beta-2-microglobulin (B2M), beta actin (ACTB), beta glucuronidase (GUSB), phosphoglycerate kinase 1 (PGK1), HGPRT (HPRT1), cyclophilin A (PPIA) and large ribosomal protein (RPLP0). Thermal cycling was carried out on a 7900HT Fast Real-Time PCR System (Applied Biosystems) according to the manufacturer’s specific settings. Data was analysed using DataAssist v3.01 (Applied Biosystems). Expression of MARCO and the endogenous control GAPDH were analysed using Taqman gene expression assays (Applied Biosystems). 50 ng/µl of RNA in 20µl reaction mix, was used for cDNA synthesis by TaqMan reverse transcription-PCR (RT-PCR) using the Verso^TM^ 2-Step QRT-PCR kit (Thermo Scientific, Surry, UK). Thermal cycling was carried out on a Stratagene MX3005P (Agilent Technologies, West Lothian, UK). Relative expression levels were determined using the ΔCt method for the basal gene expression normalizing to the endogenous controls. The fold change between macrophage populations was calculated by subtracting their ΔCt values.

**Measurement of alveolar and tissue macrophage diameter in lung resected tissue**

Sections were cut from tissue blocks and de-waxed in xylene and rehydrated in graded alcohols. Antigen retrieval was performed in a microwave in citrate buffer pH6 for 20min and slides allowed to cool for another 20min then washed in wash buffer. Slides were then blocked using 1.5% normal donkey serum for 30 minutes at room temperature an then incubated in CX_3_CR1 (Millipore, UK) primary antibody (diluted 1:800 in normal horse serum) overnight at 4°C. The next day, slides were washed in TBS-tween (0.05%) 3 times then incubated in Donkey anti-rabbit Alexa 568 (diluted 1:200 in TBS) for 90min at room temperature. After secondary antibody incubation, slides were washed in TBS-tween (0.05%) 3 times then blocked using 1.5% normal rabbit serum for 30 minutes at room temperature. Following this, slide where then incubated in either CD14 primary antibody (diluted 1:100) overnight at 4°C. The next day, slides were washed in TBS-tween (0.05%) 3 times then incubated in biotinylated rabbit anti-goat secondary antibody or goat-Anti-Rabbit IgG (diluted 1:200 in TBS) for 30min at RT. Slides were again washed in TBS-tween (0.05%) 3 times then incubated in Strep Dylight 488 (diluted 1:200 in PBS) for 1h at RT then mounted in Vectashield mountanant with DAPI (VectorLabs, Peterborough, UK). Digital micrographs were obtained using a Nikon Eclipse 80i microscope (Nikon UK Ltd, Surrey, UK) equipped with a QImaging digital camera (Media Cybernetics, Marlow UK) and cell diameters measured using ImagePro Plus 5.1 software (Media Cybernetics).

**Measurement of CXCL1 and TLR3 by Immunocytochemistry**

Cytospins stored at -20°C were defrosted for 30 min, fixed in 4% PFA for 10 min, washed and permeabilised in 0.05% Triton- X 100 (Sigma-Aldrich) in PBS for 10 min. Cells were then blocked in 2.5% normal horse blocking serum (**ImmPRESS HRP Anti-Rabbit Ig (Peroxidase) Polymer Detection Kit: Vector Labs)** for 30 min at room temperature. Cells were labelled using polyclonal rabbit anti-human CXCL1 or TLR3 primary antibodies (all from Abcam, Cambridge, UK). Primary antibody was diluted in primary antibody diluent (Diagnostic BioSystems, Pleasanton, USA) and then applied overnight at 4°C. The following day, cells were washed and then incubated in ImmPRESS™ Reagent secondary antibody (**ImmPRESS HRP Anti-Rabbit Ig (Peroxidase) Polymer Detection Kit: Vector Labs)** for 30 min at room temperature. Cells were then washed in PBS and labelled proteins were visualised by the addition of DAB HRP substrate (Vector Labs) with the reaction observed by light microscopy. The reaction was terminated by rinsing the cells with water before immersing in running water for 5 min. Cells were counterstained with Gill’s haematoxylin and Scott’s tap water then immersed in running water for 5 min. The cells were then dehydrated by immersion in a series of alcohols; 50% alcohol, 75% alcohol, 90% alcohol, 100% alcohol all for 5 min, followed by immersion in two changes of xylene for 5 min. Finally sections were mounted with a coverslip using DPX mounting medium (Sigma-Aldrich). Negative control slides were negative for DAB HRP substrate staining.

Staining was analysed using ImagePro Plus 5.1 software (Media Cybernetics). To analyse staining of small and large subpopulations by immunocytochemistry, a sizing criteria was decided. The average size of interstitial macrophages was measured to give an estimation of small macrophage size because they are predominately small. Using ImagePro Plus 5.1 software (Media Cybernetics) the average size of interstitial macrophages was calculated as 11 µms ± 5 µms (mean ± range). Therefore the criterion for small macrophages was macrophages with a mean diameter between 6 µms and 16 µms. This is in agreement with a study where they measured interstitial macrophages as 7.6 µms ± 1.8 µms . A 2 µm gap was left to provide a window to see differences between small and large macrophages. Therefore the criterion for large macrophages was macrophages with a mean diameter greater than 18 µms. A scale bar of 16µms was included in representative images to represent the maximum size of small macrophages.

The number of CXCL1+ and TLR3+ macrophages were calculated. Digital micrographs were obtained using a Nikon Eclipse 80i microscope (Nikon UK Ltd, Surrey, UK) equipped with a QImaging digital camera (Media Cybernetics, Marlow UK) and quantified using ImagePro Plus 5.1 software (Media Cybernetics). Cell counts were calculated and standardised to a total count of 200 macrophages obtained from non-consecutive frames. The number of marker positive macrophages were expressed as a percentage of the total count.

**Measurement of phagocytosis**

Enriched macrophages were re-suspended at 1.48x10^6^ macrophages/ml Uptake Buffer (HBSS with 20mM HEPES, pH 7.4). Cells were seeded at 0.1x10^6^/well (75 µl) in a non-tissue culture treated 96-well flat bottom plate (Fisher Scientific UK Ltd, Loughborough, UK) and incubated in a 5% CO_2_ humidified atmosphere at 37^o^C for 1 hour. Negative control plates were kept on ice throughout the experiment where less than 20% of macrophages carried out phagocytosis. PHrodo™ green E. coli BioParticles (Life Technologies) were added at 250 µg/ml (25 µl) and incubated in a shaking 5% CO_2_ humidified incubator at 37^o^C for 1 hour. At 1 hour, a difference in phagocytosis between small and large macrophages could be observed. Accutase® solution (Sigma-Aldrich, Dorset, UK) was added to each well (100 µl) and returned to the shaking incubator for 10 min. Using a Pasteur fine tip pipette, cells were gently scraped and collected in 5 ml tubes, washed in 2 ml PBS, re-suspended in 400 µl PBS. The cells were then acquired on BD Canto II flow cytometer setting the total acquisition to 100 000 cells for each tube. An additional gate was set around the macrophages using forward side scatter height and side scatter height to be able to gate on macrophage subpopulations. Within the macrophage subpopulation gate, an increase in FITC was proportional to phagocytosis.

#### **Phagocytosis time course**

Alveolar macrophages from 2 COPD patients and 1 smoking control patient were used to determine the optimal time for small and large macrophage phagocytosis of *E.coli* BioParticles. At 2, 3 and 4 hours both small and large macrophage phagocytosis had reached a plateau (Figure S12). At 1 hour, a difference in phagocytosis ability between small and large macrophages was observed, which was chosen as the optimal time point for future experiments.

**REFERENCES**

1. Quatromoni, J.G., et al., *Technical Advance: An optimized disaggregation method for human lung tumors that preserves the phenotype and function of the immune cells.* Journal of Leukocyte Biology, 2015. **97**(1): p. 201-209.

2. Hagman, D.K., et al., *Characterizing and quantifying leukocyte populations in human adipose tissue: Impact of enzymatic tissue processing.* Journal of Immunological Methods, 2012. **386**(1-2): p. 50-59.

3. Masten, B.J., et al., *Characterization of myeloid and plasmacytoid dendritic cells in human lung.* J Immunol, 2006. **177**(11): p. 7784-93.

4. Fuchs, H.J., J. McDowell, and J.E. Shellito, *Use of allophycocyanin allows quantitative description by flow cytometry of alveolar macrophage surface antigens present in low numbers of cells.* Am Rev Respir Dis, 1988. **138**(5): p. 1124-8.

5. Fathi, M., et al., *Functional and morphological differences between human alveolar and interstitial macrophages.* Exp Mol Pathol, 2001. **70**(2): p. 77-82.

**TABLES**

**Table S1 Subjects’ demographics for all experiments identifying macrophage subpopulations and measurement of cell surface markers by flow cytometry.**

| **Related**  **Figure / Table** | **Figure 2** | **Figure 3 /**  **Table S8** | **Table S8** |  | **Figure 3** | **Figure 4/ Table S7** | **Figure 4/ Table S7** |  | **Table S7** | **Table S7** |  | **Figure S5** |
| --- | --- | --- | --- | --- | --- | --- | --- | --- | --- | --- | --- | --- |
| **Demography** | COPD | COPD | S | P value | COPD | COPDS | COPDE | P value | COPDS | COPDE | P value | **S/NS**  **(5/2)** |
| **n** | 18 | 13 | 9 |  | 11 | 7 | 6 |  | 6 | 5 |  | 7 |
| **GOLD stage I** | 6 | 7 | N/A |  | 6 | 4 | 3 |  | 3 | 2 |  | 0 |
| **GOLD stage II** | 10 | 6 | N/A |  | 5 | 3 | 3 |  | 2 | 3 |  | 0 |
| **GOLD stage III** | 2 | 0 | N/A |  | 0 | 0 | 0 |  | 0 | 0 |  | 0 |
| **Age (yrs)** | 69.5 (6.0) | 69.4 (7.7) | 69.3 (9.4) | **0.99** | 68.7 (5.4) | 64.9 (5.1) | 74.7 (7.0) | **0.01*** | 65.8 (4.9) | 72.2 (4.0) | **0.04*** | 72 (6.0) |
| **Sex (M/F)** | 9/9 | 6/7 | 2/7 |  | 5/6 | 3/4 | 3/3 |  | 2/4 | 3/2 |  | 3/4 |
| **FEV1 (L)** | 1.6 (0.5) | 1.7 (0.5) | 2.1 (0.3) | **0.04*** | 1.7 (0.5) | 1.7 (0.4) | 1.8 (0.5) | **0.7** | 1.6 (0.5) | 1.8 (0.6) | **0.6** | 2.31 (0.5) |
| **FEV1 % Predicted** | 69.1 (14.2) | 76.0 (12.0) | 95.7 (13.8) | **0.002**** | 75.6 (11.7) | 75.6 (9.2) | 77.1 (15.6) | **0.8** | 77.0 (9.2) | 74.0 (15.2) | **0.7** | 100.8 (11.1) |
| **FVC (L)** | 2.8 (0.8) | 3.0 (0.7) | 2.7 (0.4) | **0.2** | 3.0 (0.8) | 2.9 (0.7) | 3.0 (0.8) | **0.7** | 2.8 (0.8) | 3.1 (0.8) | **0.99** | 3.09 (0.5) |
| **FEV1/FVC Ratio (%)** | 56.0 (6.6) | 57.6 (7.1) | 68.0 (25.5) | **0.3** | 56.8 (7.2) | 56.9 (7.5) | 58.4 (7.2) | **0.8** | 56.9 (8.2) | 56.8 (6.8) | **0.6** | 111.5 (15.2) |
| **Pack Year History** | 57.8 (20.4) | 55.7 (26.7) | 52.1 (15.3) | **0.7** | 62.0 (23.9) | 57.3 (29.9) | 53.8 (25.1) | **0.8** | 62.0 (23.9) | 60.0 (22.4) | **0.8** | 42.4 (20) |
| **Current smokers** | 11 | 7 | 9 |  | 6 | 7/0 | 0/0 |  | 6 | 0 |  | 5 |
| **ICS users #** | 7 | 3 | NA |  | 2 | 1 | 2 |  | 1 | 2 |  | NA |

Data shown are mean (sd). FEV1: forced expiratory volume, FVC: forced vital capacity, ICS: inhaled corticosteroid. # There were no patients on oral steroids. Unpaired t test (Two-tailed) was performed between groups. *, ** = significant difference between population (p<0.05 and 0.01 respectively)

**Table S2 Subjects’ demographics for all experiments in measurement of gene expression**

| **Related**  **Figure / Table** | **Table 2, S9, 13, 14.** | **Table S13** | **Table S14** |  | **Figure S8** | **Table S11, S12** | **Table S10** | **Table S10** |  |
| --- | --- | --- | --- | --- | --- | --- | --- | --- | --- |
| **Demography** | COPD | S | NS | P value | COPD | COPD | COPDS | COPDE | P value |
| **n** | 8 | 6 | 3 |  | 8 | 6 | 5 | 3 |  |
| **GOLD stage I** | 5 | N/A | N/A |  | 1 | 0 | 4 | 1 |  |
| **GOLD stage II** | 3 | N/A | N/A |  | 5 | 5 | 1 | 2 |  |
| **GOLD stage III** | 0 | N/A | N/A |  | 2 | 1 | 0 | 0 |  |
| **Age (yrs)** | 69.9 (5.2) | 65.2 (8.9) | 76.0 (4.4) | **0.08** | 68.3 (4.2) | 68.5 (5.8) | 67.4 (4.5) | 74.0 (3.6) | **0.07** |
| **Sex (M/F)** | 5/3 | ¼ | 2/1 |  | 4/4 | 4/2 | 2/3 | 0/3 |  |
| **FEV1 (L)** | 1.9 (0.5) | 2.1 (0.2) | 2.4 (0.4) | **0.002**** | 1.4 (0.5) | 1.5 (0.5) | 1.8 (0.5) | 2.1 (0.3) | **0.3** |
| **FEV1 % Predicted** | 79.2 (12.1) | 76.7 (33.9) | 121.0 (38.4) | **0.02*** | 60.4 (12.9) | 59.5 (10.0) | 83.1 (12.1) | 72.7 (10.8) | **0.3** |
| **FVC (L)** | 3.2 (0.8) | 2.8 (0.3) | 2.8 (0.9) | **0.8** | 2.6 (0.7) | 2.7 (0.8) | 2.9 (0.9) | 3.7 (0.4) | **0.5** |
| **FEV1/FVC Ratio (%)** | 59.8 (7.1) | 75.8 (4.7) | 76.7 (5.7) | **0.0001***** | 54.9 (7.3) | 55.5 (6.1) | 61.1 (6.5) | 57.7 (9.1) | **0.2** |
| **Pack Year History** | 65.6 (27.9) | 44.8 (18.2) | 1.7 (2.9) | **0.3 (COPD v S)** | 53.0 (6.6) | 55.0 (6.9) | 59.4 (30.3) | 76.0 (25.2) | **0.5** |
| **Current smokers** | 5 | 5/1 | N/A |  | 6 | 5 | 5 | 0 |  |
| **ICS users#** | 1 | N/A | N/A |  | 4 | 2 | 1 | 0 |  |

Data shown are mean (sd). FEV1: forced expiratory volume, FVC: forced vital capacity, ICS: inhaled corticosteroid. # There were no patients on oral steroids. Unpaired t test (Two-tailed) was performed between patient groups.*, **, *** = significant difference between patient groups (p<0.05, 0.01 and 0.001 respectively)

**Table S3 Subjects’ demographics for measurement of macrophage subpopulation diameter**

| **Related**  **Figure / Table** | **Figure 5, S9** |  |  | |  |
| --- | --- | --- | --- | --- | --- |
| **Demography** | COPDS | S | | NS | P value |
| **n** | 12 | 15 | | 11 |  |
| **GOLD stage I** | 5 | N/A | | N/A |  |
| **GOLD stage II** | 5 | N/A | | N/A |  |
| **GOLD stage III** | 2 |  | | N/A |  |
| **Age (yrs)** | 64.0 (7.3) | 65.7 (6.8) | | 69.5 (9.8) | **0.2** |
| **Sex (M/F)** | 7/5 | 9/6 | | 3/8 |  |
| **FEV1 (L)** | 1.8 (0.4) | 2.3 (0.5) | | 2.1 (0.6) | **0.03*** |
| **FEV1 % Predicted** | 68.0 (13.8) | 89.3 (13.3) | | 93.2 (18.4) | **0.0003***** |
| **FVC (L)** | 3.2 (0.8) | 3.1 (0.7) | | 2.7 (0.9) | **0.3** |
| **FEV1/FVC Ratio (%)** | 54.8 (9.1) | 75.1 (8.9) | | 73.8 (14.2) | **0.0001***** |
| **Pack Year History** | 51.2 (13.4) | 54.1 (27.6) | | N/A | **0.7** |
| **Current smokers** | 12/0 | 15/0 | | 0/0 |  |
| **ICS users#** | 5 | N/A | | N/A |  |

Data shown are mean (sd). FEV1: forced expiratory volume, FVC: forced vital capacity, ICS: inhaled corticosteroid. # There were no patients on oral steroids. Unpaired t test (Two-tailed) was performed between patient groups. *, **, *** = significant difference between patient groups (p<0.05, 0.01 and 0.001 respectively)

**Table S4 Subjects’ demographics for measurement of CXCL1 and TLR3 by immunocytochemistry**

| **Related**  **Figure / Table** | **Figure 6, S10** |
| --- | --- |
| **Demography** | COPD |
| **n** | 6 |
| **GOLD stage I** | 2 |
| **GOLD stage II** | 4 |
| **GOLD stage III** | 0 |
| **Age (yrs)** | 67.2 (5.7) |
| **Sex (M/F)** | 2/4 |
| **FEV1 (L)** | 1.6 (0.3) |
| **FEV1 % Predicted** | 73.9 (12.1) |
| **FVC (L)** | 2.8 (0.5) |
| **FEV1/FVC Ratio (%)** | 56.6 (6.9) |
| **Pack Year History** | 47.7 (19.3) |
| **Current smokers** | 3 |
| **ICS users #** | 2 |

Data shown are mean (sd). FEV1: forced expiratory volume, FVC: forced vital capacity, ICS: inhaled corticosteroid. # There were no patients on oral steroids

**Table S5 Subjects’ demographics for all experiments in measurement of phagocytosis**

| **Related**  **Figure / Table** | **Figure 6** | **Figure S11** | **Figure S11** | **Figure S11** |  |  | **Figure S12** |
| --- | --- | --- | --- | --- | --- | --- | --- |
| **Demography** | COPD | COPDS | COPDE | S | P value | COPDE | COPD and smokers |
| **n** | 9 | 6 | 3 | 6 |  | 3 | 3 |
| **GOLD stage I** | 1 | 1 | 0 | N/A |  | 1 | 0 |
| **GOLD stage II** | 6 | 4 | 2 | N/A |  | 2 | 2 |
| **GOLD stage III** | 2 | 1 | 1 | N/A |  | 0 | 0 |
| **Age (yrs)** | 68.9 (4.7) | 67.0 (4.4) | 72.7 (2.9) | 68.7 (8.5) | **0.5** | 74.0 (3.6) | 72.3 (13.1) |
| **Sex (M/F)** | 5/4 | 5/1 | 0/3 | 1/5 |  | 0/3 | 3F |
| **FEV1 (L)** | 1.5 (0.5) | 1.7 (0.4) | 1.0 (0.1) | 2.1 (0.3) | **0.003**** | 2.1 (0.3) | 2.3 (0.6) |
| **FEV1 % Predicted** | 63.1 (16.7) | 65.8 (19.5) | 57.7 (10.0) | 95.5 (16.2) | **0.01*** | 72.7 (10.8) | 68.4 (9.7) |
| **FVC (L)** | 2.6( 0.8) | 3.1(0.5) | 1.8 (0.2) | 2.6 (0.5) | **0.004**** | 3.7 (0.4) | 3.4 (1.1) |
| **FEV1/FVC Ratio (%)** | 54.9 (6.4) | 54.3 (7.3) | 56.0 (5.3) | 77.2 (3.7) | **0.0001***** | 57.7 (9.1) | 68.4 (9.7) |
| **Pack Year History** | 52.3 (8.2) | 50.0 (7.8) | 57.0 (8.0) | 49.0 (8.9) | **0.4** | 76.0 (25.2) | 42 (17.1) |
| **Current smokers** | 6 | 6/0 | 0/0 | 6/0 |  | 0 | 2 |
| **ICS users#** | 4 | 2 | 2 | N/A |  | 1 | 0 |

Data shown are mean (sd). FEV1: forced expiratory volume, FVC: forced vital capacity, ICS: inhaled corticosteroid. # There were no patients on oral steroids. Unpaired t test (Two-tailed) was performed between patient groups. *, **, *** = significant difference between patient groups (p<0.05, 0.01 and 0.001 respectively)

**Table S6 Identification of specific antibodies clones to study macrophage phenotype**.

| Marker | Clone | Isotype | Company | % cells expressing specified marker | | | |
| --- | --- | --- | --- | --- | --- | --- | --- |
|  |  |  |  | M1- like MDMs | | M2- like MDMs | |
|  |  |  |  | Monocytes+ GM-CSF | Monocytes + M-CSF+ IFN | Monocytes+ M-CSF | Monocytes+ M-CSF+IL-4 |
| CD14 | 61D3 | MsIgG1k | ebio | 29.8 | 53.7 | 49 | 7 |
| CD14 | RM052 | MsIgG2a | BC | 56.3 | 96.3 | 97.3 | 96.2 |
| CD14 | MOP9 | MsIgG2bk | BD | 51.6 | 91.9 | 96.2 | 95.6 |
| CD14 | M5E2 | MsIgG2ak | BD | 35.9 | 75.1 | 86.5 | 90.2 |
| CD38 | HB7 | Ms IgG1k | BD | 12.1 | 95.8 | 19 | 45.5 |
| CD38 | HIT2 | Ms IgG1k | ebio | 0 | 99.9 | 0 | 5.1 |
| HLA-DR | AC122 | MsIgG2ak | Miltenyi | 90.6 | 99.2 | 95.7 | 98.6 |
| HLA-DR | Immu-357 | Ms IgG1 | BC | 5.2 | 46.6 | 32.1 | 46.3 |
| HLA-DR | Tu36 | Ms IgG2bk | BD | 1.5 | 51.5 | 20.8 | 30.3 |
| HLA-DR | G46-6 | Ms IgG2ak | BD | 8.9 | 70.6 | 9.2 | 41 |
| HLA-DR | LN3 | Ms IgG2bk | ebio | 20.1 | 96.6 | 17.6 | 64.7 |
| Axl | 108724 | MsIgG1 | R+D | 58.5 | 49.9 | 89 | 46.9 |
| CD163 | GH1/61 | Ms IgG1k | ebio | 52.4 | 26.5 | 82.6 | 82 |
| CD206 | 19.2 | Ms IgG1k | ebio | 56.9 | 23.3 | 6.5 | 51.5 |
| CD206 | DCN228 | Ms IgG1k | Miltenyi | 29.7 | 9.2 | 5.8 | 43 |
| CD36 | CB38 | Ms IgMk | BD | 9.5 | 40.4 | 48.7 | 56.9 |

Peripheral blood of never smokers (n=3) was separated by Ficoll-Paque into PBMCs. MDMs were differentiated from monocytes over 12 days with either M-CSF or GM-CSF. M-CSF MDMs were further differentiated with IL-4 or IFN. Purified blood cells were single stained with antibody and the % positive cells was recorded by flow cytometry. Highlighted grey are the antibodies chosen for macrophage phenotyping experiments. Bc: Beckman Coulter, BD: BD Biosciences, eBio: eBiosciences.

**Table S7 The expression of macrophage markers in small and large macrophage populations in COPD (including smoking status)**

|  |  | **Small Alveolar Macrophages** | | | **Large Alveolar Macrophages** | | | **Small Interstitial Macrophages** | | | **Large Interstitial Macrophages** | | |
| --- | --- | --- | --- | --- | --- | --- | --- | --- | --- | --- | --- | --- | --- |
| **Marker** |  | **COPDS** | **COPDE** | **P value** | **COPDS** | **COPDE** | **P value** | **COPDS** | **COPDE** | **P value** | **COPDS** | **COPDE** | **P value** |
| **HLA-DR** | **%** | **43 (21)** | **66 (23)** | **0.097** | **22 (24)** | **60 (14)** | **0.0076 **** | **59 (28)** | **57 (30)** | **0.91** | **31 (25)** | **35 (28)** | **0.81** |
|  | **MFI** | **6 (3)** | **14 (7)** | **0.01 *** | **2 (0.3)** | **5 (0.8)** | **0.0001***** | **11 (9)** | **9 (4)** | **0.68** | **4 (5)** | **5 (0.2)** | **0.51** |
| **CD14** | **%** | **60 (17)** | **60 (23)** | **0.98** | **3 (3)** | **20 (12)** | **0.0026 **** | **51 (12)** | **51 (29)** | **0.97** | **3 (3)** | **14 (16)** | **0.097** |
|  | **MFI** | **15 (8)** | **32 (35)** | **0.20** | **1 (0.1)** | **2 (0.1)** | **0.0001***** | **11 (15)** | **13 (2)** | **0.86** | **1 (0.1)** | **1 (0.1)** | **0.48** |
| **CD38** | **%** | **56 (12)** | **70 (22)** | **0.15** | **3 (4)** | **20 (14)** | **0.0064 **** | **60 (10)** | **61 (16)** | **0.84** | **5 (9)** | **16 (14)** | **0.17** |
|  | **MFI** | **7 (2)** | **12 (8)** | **0.10** | **1 (0.1)** | **2 (0.3)** | **0.0001***** | **8 (3)** | **9 (1)** | **0.59** | **1 (0.5)** | **2 (0.3)** | **0.041*** |
| **CD36** | **%** | **52 (17)** | **71 (15)** | **0.066** | **2 (2)** | **20 (34)** | **0.14** | **48 (15)** | **55 (14)** | **0.45** | **1 (1)** | **10 (8)** | **0.011*** |
|  | **MFI** | **9 (6)** | **18 (15)** | **0.13** | **1 (0.1)** | **1 (0.1)** | **0.0001***** | **10 (7)** | **8 (8)** | **0.79** | **1 (0.1)** | **0.6 (0.6)** | **0.077** |
| **CD206** | **%** | **12 (2)** | **10 (5)** | **0.40** | **57 (21)** | **86 (11)** | **0.016 *** | **15 (5)** | **12 (5)** | **0.36** | **54 (22)** | **59 (31)** | **0.74** |
|  | **MFI** | **1 (0.2)** | **1 (0.1)** | **0.14** | **3 (0.4)** | **22 (6)** | **0.0001***** | **1.3 (0.2)** | **0.98 (0.05)** | **0.0052**** | **9 (13)** | **23 (10)** | **0.11** |
| **CD163** | **%** | **20 (8)** | **24 (17)** | **0.59** | **8 (11)** | **53 (19)** | **0.0002***** | **11 (5)** | **9 (5)** | **0.63** | **9 (16)** | **30 (30)** | **0.14** |
|  | **MFI** | **2 (0.7)** | **2 (0.9)** | **0.36** | **1 (0.3)** | **1 (0.5)** | **0.0001***** | **1.5 (0.2)** | **1 (0.1)** | **0.014*** | **2 (0.9)** | **5 (3)** | **0.012*** |

Flow cytometric analysis of macrophage subpopulations for COPD smokers (COPDS) (n=7 alveolar macrophages and n=6 interstitial macrophages) and COPD ex-smokers (COPDE) (n=6 alveolar macrophages and n=5 interstitial macrophages). Data is expressed as the percentage of cells within each subpopulation expressing specific marker (%) and median fluorescence intensity (MFI) and represents mean (SEM). Unpaired t test (Two-tailed) was performed for each marker.

*, **, ***= significantly different expression compared to macrophages from COPDE patients (p<0.05, 0.01 and 0.001 respectively).

**Table S8 The expression of macrophage markers in small and large macrophages from COPD patients compared to smoking controls**

|  |  | **Small Alveolar Macrophages** | | | **Large Alveolar Macrophages** | | |
| --- | --- | --- | --- | --- | --- | --- | --- |
| **Marker** |  | **COPD** | **S** | **P value** | **COPD** | **S** | **P value** |
| **HLA-DR** | **%** | **52 (24)** | **50 (27)** | **0.87** | **36 (28)** | **23 (16)** | **0.23** |
|  | **MFI** | **9 (6)** | **9 (8)** | **0.96** | **3 (2)** | **2 (0.5)** | **0.13** |
| **CD14** | **%** | **60 (19)** | **64 (16)** | **0.61** | **9 (12)** | **5 (4)** | **0.34** |
|  | **MFI** | **22 (23)** | **24 (7)** | **0.80** | **1 (0.3)** | **1 (0.1)** | **0.48** |
| **CD38** | **%** | **61 (17)** | **58 (10)** | **0.62** | **9 (12)** | **5 (4)** | **0.37** |
|  | **MFI** | **9 (6)** | **8 (2)** | **0.73** | **1 (0.4)** | **1 (0.1)** | **0.54** |
| **CD36** | **%** | **59 (18)** | **58 (15)** | **0.87** | **9 (22)** | **7 (6)** | **0.77** |
|  | **MFI** | **13 (11)** | **12 (7)** | **0.98** | **1 (0.1)** | **1 (0.5)** | **0.27** |
| **CD206** | **%** | **11 (3)** | **26 (15)** | **0.0021**** | **68 (23)** | **66 (25)** | **0.83** |
|  | **MFI** | **1 (0.2)** | **2 (2)** | **0.023 *** | **10 (10)** | **5 (2)** | **0.12** |
| **CD163** | **%** | **22 (12)** | **23 (13)** | **0.74** | **26 (27)** | **20 (20)** | **0.63** |
|  | **MFI** | **2 (0.8)** | **3 (1)** | **0.33** | **3 (2)** | **3 (3)** | **0.86** |

Flow cytometric analysis of macrophage subpopulations for n=13 COPD and n=9 smoking controls (S). Data is expressed as the percentage of cells within each subpopulation expressing specific marker (%) and median fluorescence intensity (MFI) and represents mean (SEM). Unpaired t test (Two-tailed) was performed for each marker.

*, **, ***= significantly different expression compared to macrophages from COPDE patients (p<0.05, 0.01 and 0.001 respectively).

**Table S9 Inflammatory related genes that are significantly expressed in interstitial macrophages compared to alveolar macrophages from COPD patients.**

| **Gene name** | **Gene ID** | ***P* Value** | **Fold change** | **Function** |
| --- | --- | --- | --- | --- |
| NLRP3 | Hs00918082_m1 | 0.0009 | 20.08 | Inflammation /// immune response /// apoptosis |
| IL1B | Hs01555410_m1 | 0.0039 | 16.19 | Cytokine /// inflammation /// cell proliferation and differentiation /// apoptosis |
| IL1RL1 | Hs00545033_m1 | 0.0004 | 15.76 | Cytokine receptor /// pro-inflammatory |
| IL6 | Hs00985639_m1 | 0.0016 | 15.48 | Cytokine /// pro and anti-inflammatory |
| SFTPD | Hs00358340_m1 | 0.0002 | 13.62 | Surfactant protein /// host defence |
| IL10 | Hs00961622_m1 | 0.0002 | 13.59 | Cytokine /// immunoregulatory |
| MUC5B | Hs00861595_m1 | 0.0002 | 12.97 | Mucin /// host defence |
| SFTPA1 | Hs00831305_s1 | 0.0005 | 12.80 | Surfactant protein /// host defence |
| CXCL1 | Hs00605382_g1 | 0.0001 | 12.19 | Growth factor /// neutrophil chemoattractant |
| MUC1 | Hs00159357_m1 | 0.0001 | 12.11 | Mucin /// host defence |
| LTF | Hs00914334_m1 | 0.0040 | 11.33 | Transferrin /// iron homeostasis /// host defence /// anti-inflammatory |
| MUC5AC | Hs00873651_m1 | 0.0034 | 10.74 | Mucin /// host defence |
| CCL20 | Hs01011368_m1 | 0.0074 | 9.84 | Lymphocyte chemoattractant |
| CCL3 | Hs00234142_m1 | 0.0034 | 9.20 | Acute inflammation /// granulocyte recruitment |
| SLPI | Hs00268204_m1 | 0.0001 | 9.11 | Serum protease inhibitor /// Prevents epithelium degradation |
| SERPINE1 | Hs01126606_m1 | 0.0046 | 8.82 | Fibrinolysis inhibitor |
| DUOX1 | Hs00213694_m1 | 0.0019 | 8.46 | Generates hydrogen peroxide /// antimicrobial defence |
| CSF3 | Hs00738432_g1 | 0.0029 | 8.44 | Cytokine /// granulocyte activation and differentiation |
| ICAM1 | Hs00164932_m1 | 0.0002 | 7.35 | Adhesion /// transmigration |
| CLDN1 | Hs00221623_m1 | 0.0032 | 7.23 | Integral membrane protein /// tight junction strands ///cell-to-cell adhesion// |
| VEGFA | Hs00900055_m1 | 0.0006 | 6.50 | Growth factor /// angiogenesis /// cell migration /// apoptosis inhibition |
| HSPA1A | Hs00359163_s1 | 0.0008 | 5.96 | Protein stabilization /// folding of newly translated proteins /// ubiquitin-proteasome pathway |
| TJP1 | Hs01551861_m1 | 0.0007 | 5.95 | Signal transduction at cell-cell junctions |
| IL1A | Hs00174092_m1 | 0.0075 | 5.74 | Cytokine /// pro-inflammatory /// apoptosis induction |
| OCLN | Hs00170162_m1 | 0.0006 | 5.44 | Tight junction permeability barrier regulation |
| SOD2 | Hs00167309_m1 | 0.0005 | 5.09 | Hydrogen peroxide generation |
| IL1R2 | Hs01030384_m1 | 0.0096 | 4.92 | Decoy receptor /// inhibitory signal /// anti-inflammatory |
| INHBB | Hs00173582_m1 | 0.0015 | 4.82 | Inhibin /// negative regulator of cell proliferation /// tumour-suppressor |
| CRLF2 | Hs00845692_m1 | 0.0082 | 4.47 | Cell proliferation /// hematopoietic system development |
| BMP4 | Hs00370078_m1 | 0.0004 | 4.40 | Cell proliferation /// bone formation |
| IL1R1 | Hs00991002_m1 | 0.0006 | 4.18 | Cytokine receptor /// pro-inflammatory |
| PI3 | Hs00160066_m1 | 0.0026 | 3.76 | Antimicrobial peptide |
| TGFB3 | Hs01086000_m1 | 0.0065 | 3.43 | Cell proliferation |
| TGFB2 | Hs00234244_m1 | 0.0023 | 3.38 | Cell proliferation |
| CD200 | Hs01033303_m1 | 0.0050 | 3.35 | Macrophage lineage inhibitory signal |
| TLR2 | Hs01872448_s1 | 0.0008 | 3.22 | Host response to gram-positive bacteria and yeast |
| NQO1 | Hs02512143_s1 | 0.0016 | 3.20 | Prevents production of ROS |
| BMP7 | Hs00233476_m1 | 0.0063 | 3.16 | Bone growth |
| DEFB4A;DEFB4B | Hs00175474_m1 | 0.0066 | 3.16 | Antibiotic peptide |
| TLR3 | Hs01551078_m1 | 0.0043 | 3.14 | Host defence against viruses |
| IFNB1 | Hs01077958_s1 | 0.0063 | 3.07 | Antiviral /// antibacterial /// anticancerous |
| CCL11 | Hs00237013_m1 | 0.0067 | 3.06 | Eosinophil chemoattractant |
| CCL26 | Hs00171146_m1 | 0.0067 | 3.06 | Eosinophil and basophil chemoattractant |
| CSF2 | Hs00929873_m1 | 0.0067 | 3.06 | Granulocyte and macrophage production /// differentiation //// activation |
| IFNA1 | Hs00855471_g1 | 0.0067 | 3.06 | Cytokine /// antiviral |
| IFNA2 | Hs00265051_s1 | 0.0067 | 3.06 | Cytokine /// antiviral |
| IFNA4 | Hs01681284_sH | 0.0067 | 3.06 | Cytokine /// antiviral |
| IL12A | Hs01073447_m1 | 0.0067 | 3.06 | Cytokine /// IFNγ induction /// Th1 and Th2 cell differentiation |
| IL12RB2 | Hs01548202_m1 | 0.0067 | 3.06 | Cytokine receptor /// T cell differentiation |
| IL13 | Hs00174379_m1 | 0.0067 | 3.06 | Cytokine /// immunoregulatory |
| IL13RA2 | Hs00152924_m1 | 0.0067 | 3.06 | Cytokine receptor /// IL13 internalisation |
| IL28A | Hs00820125_g1 | 0.0067 | 3.06 | Cytokine /// antiviral |
| IL29 | Hs00601677_g1 | 0.0067 | 3.06 | Cytokine /// antiviral |
| INHA | Hs00171410_m1 | 0.0067 | 3.06 | Inhibin /// cell proliferation /// apoptosis /// immune response /// hormone secretion |
| LPO | Hs00976400_m1 | 0.0067 | 3.06 | Oxidoreductase /// Antibacterial agent |
| MBL2 | Hs00175093_m1 | 0.0067 | 3.06 | Collectin /// innate immune system // classical complement pathway |
| NOS2 | Hs01075529_m1 | 0.0067 | 3.06 | Free radical /// neurotransmission /// antimicrobial /// antitumor activities |
| IL18R1 | Hs00977691_m1 | 0.0070 | 3.00 | Cytokine receptor /// pro-inflammatory |
| DUOX2 | Hs00204187_m1 | 0.0038 | 2.94 | ROS production /// cell signalling /// cell damage |
| ACVR2A | Hs00155658_m1 | 0.0006 | 2.88 | Activin /// cell proliferation /// differentiation /// apoptosis /// immune response |
| SIGIRR | Hs00222347_m1 | 0.0038 | 2.51 | Negative modulator /// anti-inflammatory |
| NFKB1 | Hs00765730_m1 | 0.0063 | 2.12 | Transcription factor /// inhibitory |
| BMP1 | Hs00241807_m1 | 0.0016 | 1.84 | Cell proliferation /// cartilage formation |
| NLRC4 | Hs00892666_m1 | 0.0055 | 0.65 | Inflammasome formation /// activated by bacteria |
| OAS1 | Hs00973637_m1 | 0.0022 | 0.63 | Innate immune response /// anti-viral |
| ALOX5 | Hs01095330_m1 | 0.0013 | 0.54 | Leukotriene synthesis |
| INHBA | Hs01081598_m1 | 0.0093 | 0.41 | Inhibin and activin /// negative regulation of cell proliferation |
|  |  |  |  |  |

Macrophage mRNA was analysed using a Custom Made Taqman Assay and data was analysed with alveolar macrophages as the reference. Genes with > 1.5 fold change or < 1.5 fold change are shown. Paired t test (two tailed) was performed and p<0.01 for all genes.

**Table S10 Inflammatory related genes that are significantly expressed in alveolar macrophages from COPD smokers compared to COPD ex-smokers**

| Gene name | Gene ID | *P* value | Fold change | Function |
| --- | --- | --- | --- | --- |
| GCLC | Hs00155249_m1 | 0.0036 | 2.70 | Glutathione synthesis /// antioxidant |
| PTAFR | Hs00265399_s1 | 0.0011 | 1.55 | GPCR /// angiogenesis /// pro-inflammatory |
| IFNG | Hs00989291_m1 | <0.0001 | 0.19 | Antiviral |
| IL1B | Hs01555410_m1 | 0.0034 | 0.15 | Pro-inflammatory cytokine |
| CAMP | Hs00189038_m1 | 0.0085 | 0.13 | Second messenger /// signal transduction |
| TNF | Hs01113624_g1 | 0.0014 | 0.08 | Pro-inflammatory cytokine |

Macrophage mRNA was analysed using a Custom Made Taqman Assay. Data was analysed with COPD ex-smoker alveolar macrophages as the reference. Genes with > 1.5 fold change or < 1.5 fold change are shown. Unpaired t test (two tailed) was performed and p<0.01 for all genes.

**Table S11 Inflammatory related genes that are significantly expressed in small interstitial macrophages compared to small alveolar macrophages from COPD patients.**

| **Gene name** | **Gene ID** | ***P* Value** | **Fold change** | **Function** |
| --- | --- | --- | --- | --- |
| CCL20 | Hs01011368_m1 | 0.0050 | 114.21 | Lymphocyte chemoattractant |
| CXCL1 | Hs00605382_gH | 0.0018 | 16.56 | Growth factor /// neutrophil chemoattractant |
| LTF | Hs00914334_m1 | 0.0026 | 11.91 | Transferrin /// iron homeostasis /// host defence /// anti-inflammatory |
| INHBB | Hs00173582_m1 | 0.0006 | 11.06 | Inhibin /// negative regulator of cell proliferation /// tumour-suppressor |
| CCL3 | Hs00234142_m1 | 0.0073 | 10.63 | Acute inflammation /// granulocyte recruitment |
| MUC5B | Hs00861595_m1 | 0.0011 | 10.09 | Mucin /// host defence |
| ICAM1 | Hs00164932_m1 | 0.0063 | 9.77 | Adhesion /// transmigration |
| VEGFA | Hs00900055_m1 | 0.0066 | 6.26 | Growth factor /// angiogenesis /// cell migration /// apoptosis inhibition |
| IL1R1 | Hs00991002_m1 | 0.0061 | 5.44 | Cytokine receptor /// pro-inflammatory |
| TLR3 | Hs01551078_m1 | 0.0093 | 5.36 | Host defence against viruses |
| MUC5AC | Hs00873651_mH | 0.0007 | 4.92 | Mucin /// host defence |
| IGF1 | Hs01547656_m1 | 0.0023 | 4.83 | Stimulates cell growth /// proliferation /// inhibits programmed cell death |
| TGFB2 | Hs00234244_m1 | 0.0097 | 4.42 | Cell proliferation |
| IL1RL1 | Hs00545033_m1 | 0.0075 | 3.42 | Cytokine receptor /// pro-inflammatory |
| ACVR2A | Hs00155658_m1 | 0.0096 | 3.21 | Activin /// cell proliferation /// differentiation /// apoptosis /// immune response |
| NQO1 | Hs02512143_s1 | 0.0036 | 3.07 | Prevents production of ROS |
| NFE2L2 | Hs00975960_m1 | 0.0024 | 2.50 | Transcription factor /// antioxidant |

Macrophage mRNA was analysed using a Custom Made Taqman Assay and data was analysed with small alveolar macrophages as the reference. Genes with > 1.5 fold change or < 1.5 fold change are shown. Paired t test (two tailed) was performed and p<0.01 for all genes.

**Table S12 Inflammatory related genes that are significantly expressed in large alveolar macrophages compared to small alveolar macrophages from COPD patients.**

| **Gene name** | **Gene ID** | ***P* Value** | **Fold change** | **Function** |
| --- | --- | --- | --- | --- |
| INHBA | Hs01081598_m1 | 0.0005 | 31.31 | Inhibin and activin /// negative regulation of cell proliferation |
| CXCL5 | Hs00171085_m1 | 0.0030 | 24.42 | Neutrophil chemotaxis |
| IL1A | Hs00174092_m1 | 0.0075 | 6.24 | Cytokine /// pro-inflammatory /// apoptosis induction |
| ALOX5 | Hs01095330_m1 | 0.0006 | 3.52 | Leukotriene synthesis |
| PRDX1 | Hs00602020_mH | 0.0024 | 2.95 | Antioxidant /// antiviral |
| SERPINA1 | Hs01097800_m1 | 0.0048 | 2.14 | Alpha-1 antitrypsin production /// inhibits neutrophil elastase |
| IFIH1 | Hs01070332_m1 | 0.0098 | 1.76 | PRR /// host defence against viruses |
| CAT | Hs00156308_m1 | 0.0016 | 1.69 | Antioxidant |
| MX1 | Hs00895608_m1 | 0.002 | 1.63 | Antiviral |
| SOD1 | Hs00533490_m1 | 0.0055 | 1.62 | Superoxide dismutase /// antioxidant |
| EIF2AK2 | Hs00169345_m1 | 0.0002 | 1.55 | Antiviral |
| ACTB | Hs99999903_m1 | 0.0006 | 0.65 | Actin /// cell motility /// structure /// integrity |
| MAPK3 | Hs00385075_m1 | 0.0078 | 0.64 | kinase /// signalling cascade /// proliferation /// differentiation |
| IRF7 | Hs01014809_g1 | 0.0016 | 0.59 | Transcriptional activation of IFNs /// antiviral |
| IL15 | Hs01003716_m1 | 0.0078 | 0.56 | Cytokine /// antiviral |
| TLR5 | Hs01920773_s1 | 0.0093 | 0.55 | PRR /// recognizes flagellin |
| TP53 | Hs01034249_m1 | 0.0007 | 0.53 | Cell cycle arrest /// apoptosis /// senescence /// DNA repair |
| NOD2 | Hs00223394_m1 | 0.0041 | 0.52 | PRR /// recognizes bacteria /// host defence |
| CD14 | Hs02621496_s1 | 0.0003 | 0.51 | PRR /// recognizes LPS |
| NFKB1 | Hs00765730_m1 | 0.0011 | 0.49 | Transcription factor /// inhibitory |
| FOS | Hs99999140_m1 | 0.0067 | 0.43 | Transcription factor |
| IL16 | Hs00189606_m1 | 0.0003 | 0.42 | Cytokine /// chemoattractant /// T cell activation modulator |
| IFNAR2 | Hs01022060_m1 | 0.0023 | 0.42 | Cytokine receptor |
| HGF | Hs00300159_m1 | 0.0021 | 0.39 | Growth factor /// motility /// morphogenic factor |
| ICAM1 | Hs00164932_m1 | 0.0073 | 0.35 | Adhesion /// transmigration |
| S100A9 | Hs00610058_m1 | 0.0028 | 0.31 | Cell cycle progression /// cell differentiation |
| IL10 | Hs00961622_m1 | 0.0021 | 0.29 | Cytokine /// immunoregulatory |
| IL1R1 | Hs00991002_m1 | 0.0089 | 0.29 | Cytokine receptor /// pro- inflammatory |
| S100A8 | Hs00374264_g1 | 0.0062 | 0.25 | Cell cycle progression /// cell differentiation |
| SIGIRR | Hs00222347_m1 | 0.0007 | 0.24 | Negative modulator /// anti-inflammatory |
| MUC1 | Hs00159357_m1 | 0.0086 | 0.24 | Mucin /// host defence |
| CCL5 | Hs00174575_m1 | 0.0077 | 0.22 | T cells, eosinophil and basophil chemotaxis |
| IL1RL1 | Hs00545033_m1 | 0.0015 | 0.16 | Cytokine receptor /// pro-inflammatory |
| SLPI | Hs00268204_m1 | 0.0009 | 0.16 | Serum protease inhibitor /// Prevents epithelium degradation |
| VEGFA | Hs00900055_m1 | 0.0006 | 0.15 | Growth factor /// angiogenesis /// cell migration /// apoptosis inhibition |
| SFTPD | Hs00358340_m1 | 0.0042 | 0.14 | Surfactant protein /// host defence |
| CCL17 | Hs00171074_m1 | 0.0007 | 0.14 | T cell chemotaxis |
| TLR2 | Hs01872448_s1 | <0.0001 | 0.13 | Host response to Gram-positive bacteria and yeast |
| NLRP3 | Hs00918082_m1 | 0.0004 | 0.11 | Inflammation /// immune response /// apoptosis |
| IL1R2 | Hs01030384_m1 | 0.0007 | 0.10 | Decoy receptor /// inhibitory signal /// anti-inflammatory |
| SFTPA1 | Hs00831305_s1 | 0.0006 | 0.07 | Surfactant protein /// host defence |
| CCL22 | Hs01574247_m1 | <0.0001 | 0.06 | Chemokine /// trafficking of activated T lymphocytes to inflammatory sites |

Macrophage mRNA was analysed using a Custom Made Taqman Assay and data was analysed with small alveolar macrophages as the reference. Genes with > 1.5 fold change or < 1.5 fold change are shown. Paired t test (two tailed) was performed and p<0.01 for all genes.

**Table S13 Inflammatory related genes that are significantly expressed in COPD alveolar macrophages compared to smoker alveolar macrophages.**

| Gene name | Gene ID | P value | Fold change | Function |
| --- | --- | --- | --- | --- |
| MAPK8 | Hs00177083_m1 | 0.0017 | 2.28 | kinase /// cell proliferation /// differentiation /// migration /// programmed cell death |
| TRAF6 | Hs00371512_g1 | 0.0024 | 1.93 | signal transduction |
| BMPR2 | Hs00176148_m1 | 0.0008 | 1.80 | kinase /// cell growth ///cell differentiation |
| FADD | Hs04187499_m1 | 0.0037 | 1.67 | adaptor protein /// apoptosis |
| ALOX5 | Hs01095330_m1 | 0.0030 | 1.58 | Leukotriene synthesis |

Macrophage mRNA was analysed using a Custom Made Taqman Assay. Data was analysed with smoker alveolar macrophages as the reference. Genes with > 1.5 fold change or < 1.5 fold change are shown. Paired t test (two tailed) was performed and p<0.01 for all genes.

**Table S14 Inflammatory related genes that are significantly expressed in COPD alveolar macrophages compared to never smoker alveolar macrophages.**

| Gene name | Gene ID | P value | Fold change | Function |
| --- | --- | --- | --- | --- |
| CYBB | Hs00166163_m1 | 0.0084 | 5.81 | ROS production |
| IFNAR1 | Hs01066118_m1 | 0.0034 | 5.19 | Antiviral |
| OAS2 | Hs00942643_m1 | 0.0013 | 4.33 | Antiviral |
| DDX58 | Hs00204833_m1 | 0.0013 | 3.75 | PRR /// antiviral |
| FAS | Hs00236330_m1 | 0.0093 | 3.70 | Apoptosis |
| OAS1 | Hs00973637_m1 | 0.0003 | 3.55 | Antiviral |
| ISG15 | Hs01921425_s1 | 0.0059 | 3.04 | Ubiquitination |
| ADAR | Hs00241666_m1 | 0.0015 | 2.95 | RNA- editing |
| MAPK8 | Hs00177083_m1 | 0.0005 | 2.90 | Kinase /// cell proliferation /// differentiation /// migration /// programmed cell death |
| ALOX5 | Hs01095330_m1 | 0.0023 | 2.83 | Leukotriene synthesis |
| BMPR2 | Hs00176148_m1 | 0.0019 | 2.70 | kinase /// cell growth ///cell differentiation |
| TLR7 | Hs01933259_s1 | 0.0010 | 2.69 | PRR /// recognises ssRNA in endosomes /// antiviral |
| EIF2AK2 | Hs00169345_m1 | 0.0045 | 2.60 | PRR /// recognises ssRNA in endosomes /// antiviral |
| MAPK3 | Hs00385075_m1 | 0.0006 | 2.57 | Antiviral |
| GSR | Hs00167317_m1 | 0.0041 | 2.51 | Oxidative stress prevention /// scavenger |
| PTAFR | Hs00265399_s1 | 0.0029 | 2.33 | GPCR /// angiogenesis /// pro-inflammatory |
| TICAM2 | Hs04189225_m1 | 0.0064 | 2.31 | Adaptor protein /// TLR signalling |
| CASP8 | Hs01018151_m1 | 0.0064 | 2.20 | Apoptosis |
| HSPA5 | Hs00607129_gH | 0.0061 | 2.16 | Chaperone /// protein translocation and folding |
| TRAF6 | Hs00371512_g1 | 0.0038 | 2.11 | Signal transduction |
| NFE2L2 | Hs00975960_m1 | 0.0072 | 2.07 | Transcription factor /// antioxidant |
| PYCARD | Hs01547324_gH | 0.0096 | 2.01 | Adaptor protein /// apoptosis |
| TP53 | Hs01034249_m1 | 0.0070 | 1.86 | Cell cycle arrest /// apoptosis /// senescence /// DNA repair |
| MAPK14 | Hs00176247_m1 | 0.0042 | 1.84 | Kinase /// signalling cascade /// proliferation /// differentiation |
| GSTP1 | Hs02512067_s1 | 0.0090 | 0.41 | Detoxification |
| ACVR1 | Hs00153836_m1 | 0.0056 | 0.39 | Activin // growth /// differentiation |
| SOD1 | Hs00533490_m1 | 0.0091 | 0.23 | Superoxide dismutase /// antioxidant |
| TOLLIP | Hs01553188_m1 | 0.0032 | 0.17 | Inhibitory adaptor protein /// TLR signalling |
| PRDX1 | Hs00602020_mH | 0.0038 | 0.16 | Antioxidant /// antiviral |

Macrophage mRNA was analysed using a Custom Made Taqman Assay. Data was analysed with never smoker alveolar macrophages as the reference. Genes with > 1.5 fold change or < 1.5 fold change are shown. Unpaired t test (two tailed) was performed and p<0.01 for all genes.

**
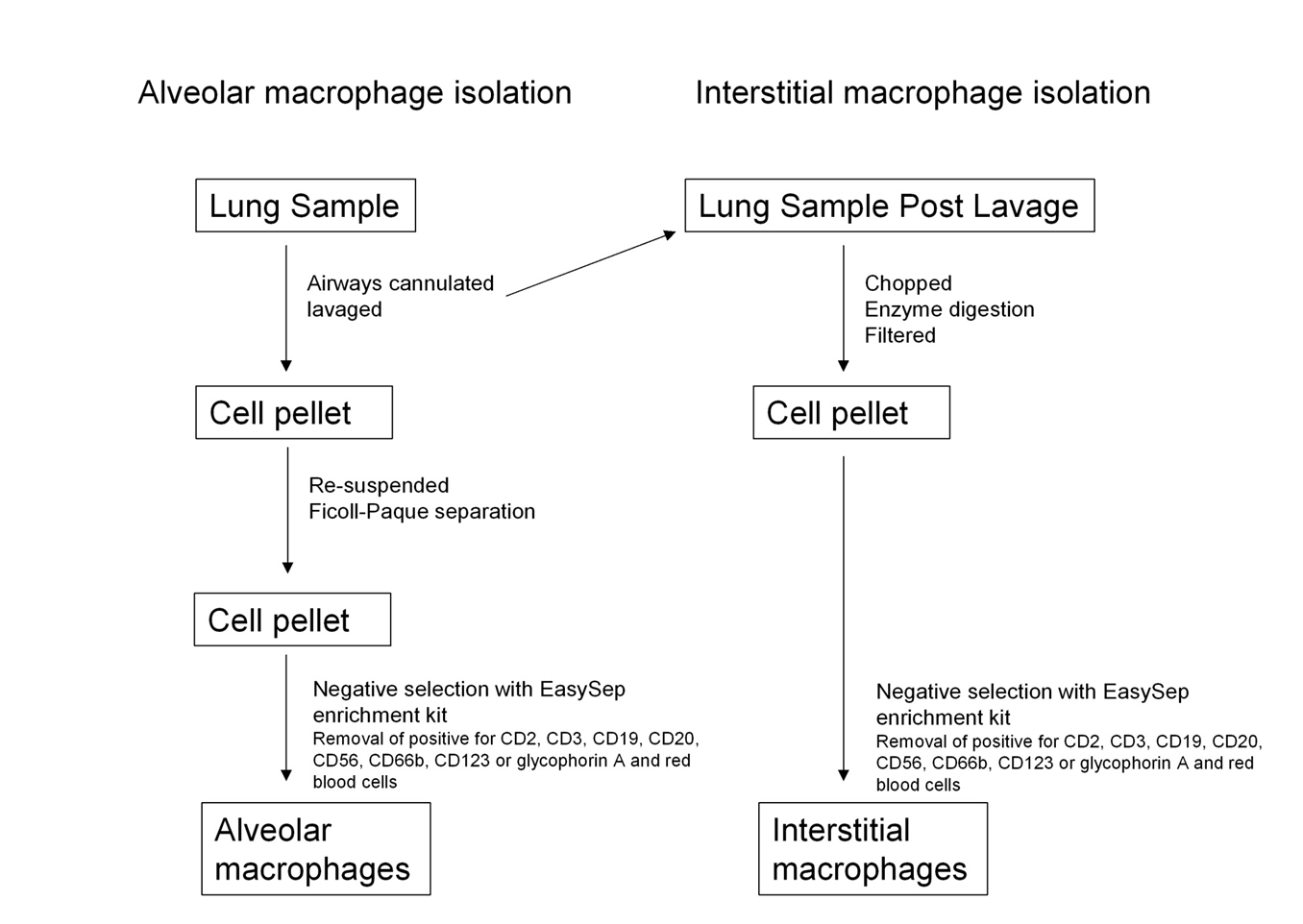
**

**SUPPLEMTARY FIGURES**

**Figure S1 Flow diagram of isolation of alveolar and interstitial macrophages from resected lung tissue.**


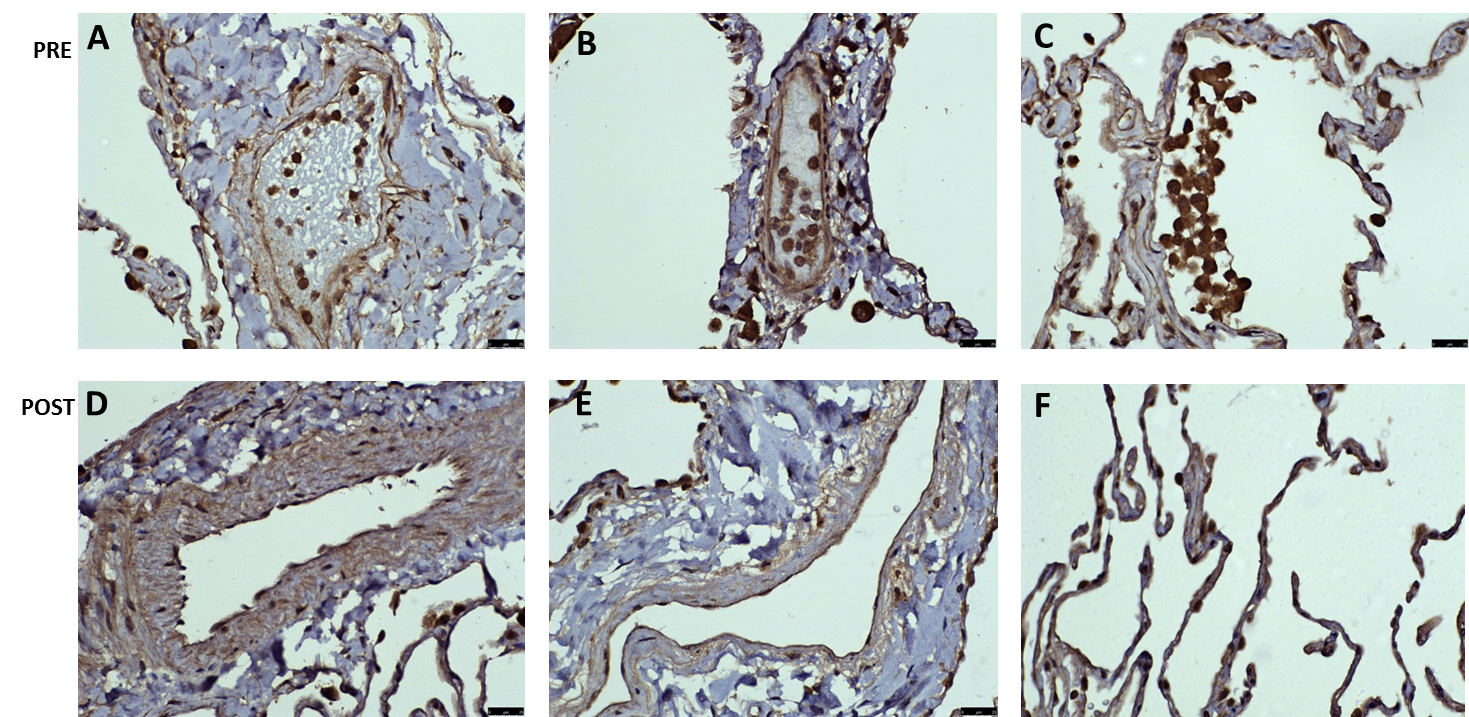
**Figure S2 Immunohistochemistry of pre- and post-flushed lung tissue for the monocyte/macrophage marker CX_3_CR1**.

FFPE resected lung tissue pre- (A-C) and post-flushing (D-F) were stained for the monocyte/macrophage marker CX_3_CR1. Blood monocyte (CX_3_CR1 positive cells in blood vessels) are shown to be present in pre-flush (A-B) and absent post-flush (D-E) tissue. Alveolar macrophages (CX_3_CR1 positive cells in alveolar space) are shown to be present in pre-flush (C) and absent post-flush (F) tissue.

**Figure S3 Differential cell counts of alveolar and tissue cells isolated from resected lung tissue pre and post enrichment using EasySep monocyte enrichment cocktail.**


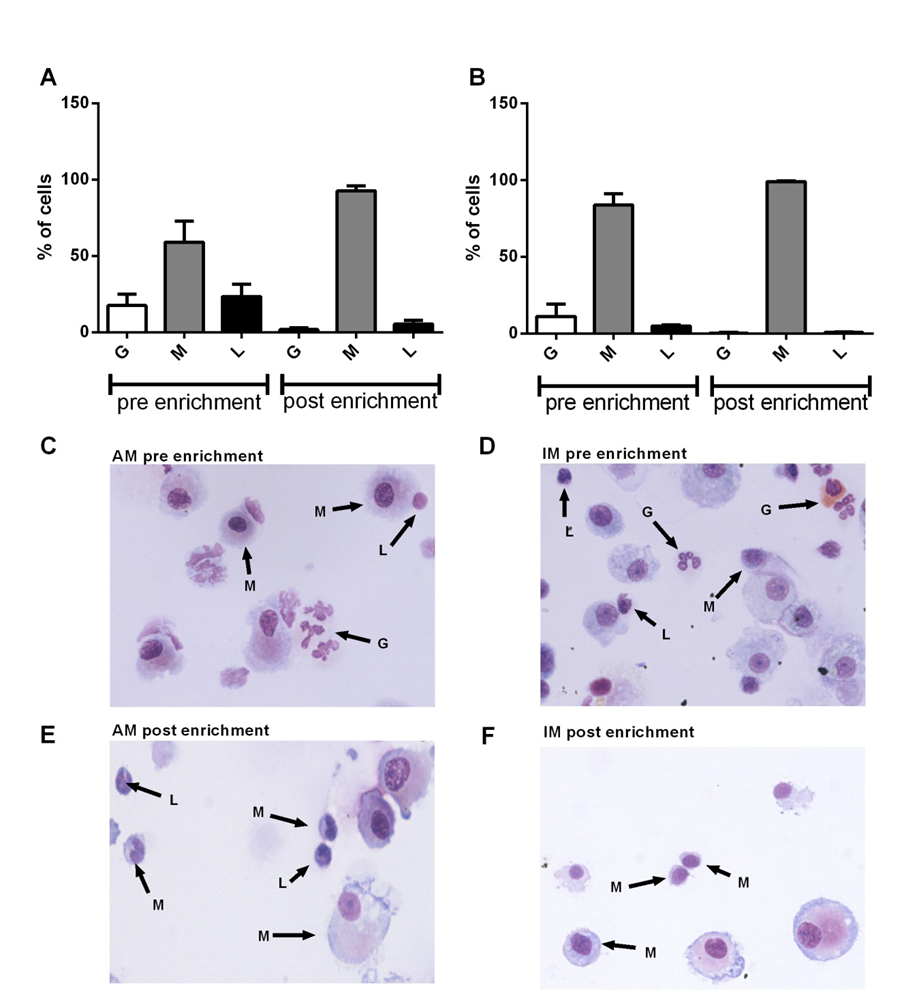
Cytospins of alveolar cells (A; n=4) and tissue cells (B; n=3) were stained with RapiDiff and 400 cells were counted. The number of granulocytes (G), macrophages (M) and lymphocytes (L) are expressed as a percentage of the total count. Data shown are mean (SEM). Representative images of RapiDiff stained cytospin slides shown for alveolar macrophages pre and post enrichment (C and E respectively) and interstitial macrophages pre and post enrichment (D and F respectively). Arrows used to show granulocytes (G), macrophages (M) and lymphocytes (L).


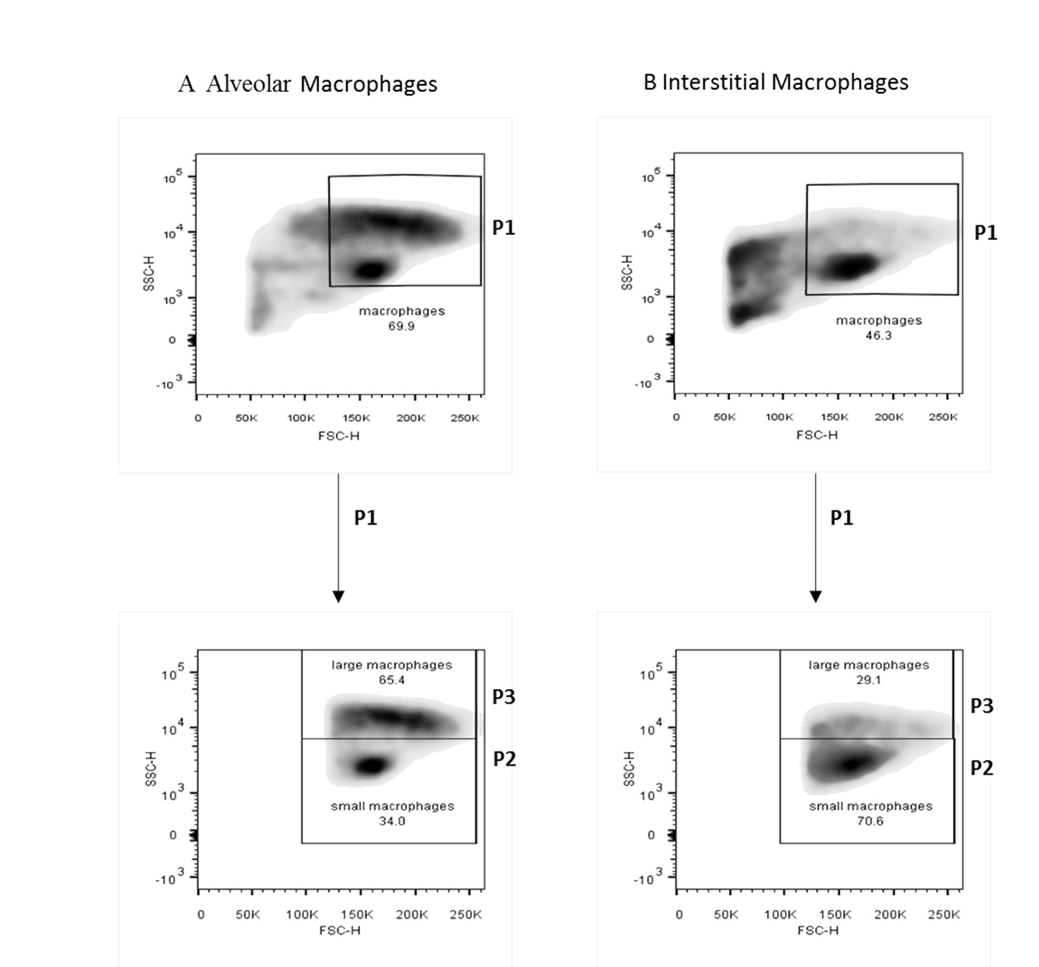


**Figure S4 Gating strategy for studying macrophage subpopulations by flow cytometry.** Alveolar (A) and interstitial (B) macrophages were isolated following EasySep purification (upper panel). Events less than 100k FSC-H were classed as debris. To exclude debris a gate was drawn around events greater than 100k FSC-H (P1 upper panel). Only events within this gate P1 were analysed further (bottom panel) as macrophage subpopulations for either marker expression or proportions of subpopulations. P1 represent whole macrophage population. P2 represent small macrophages and P3 represent large macrophages, P2 and P3 are subpopulations of P1 gated events.


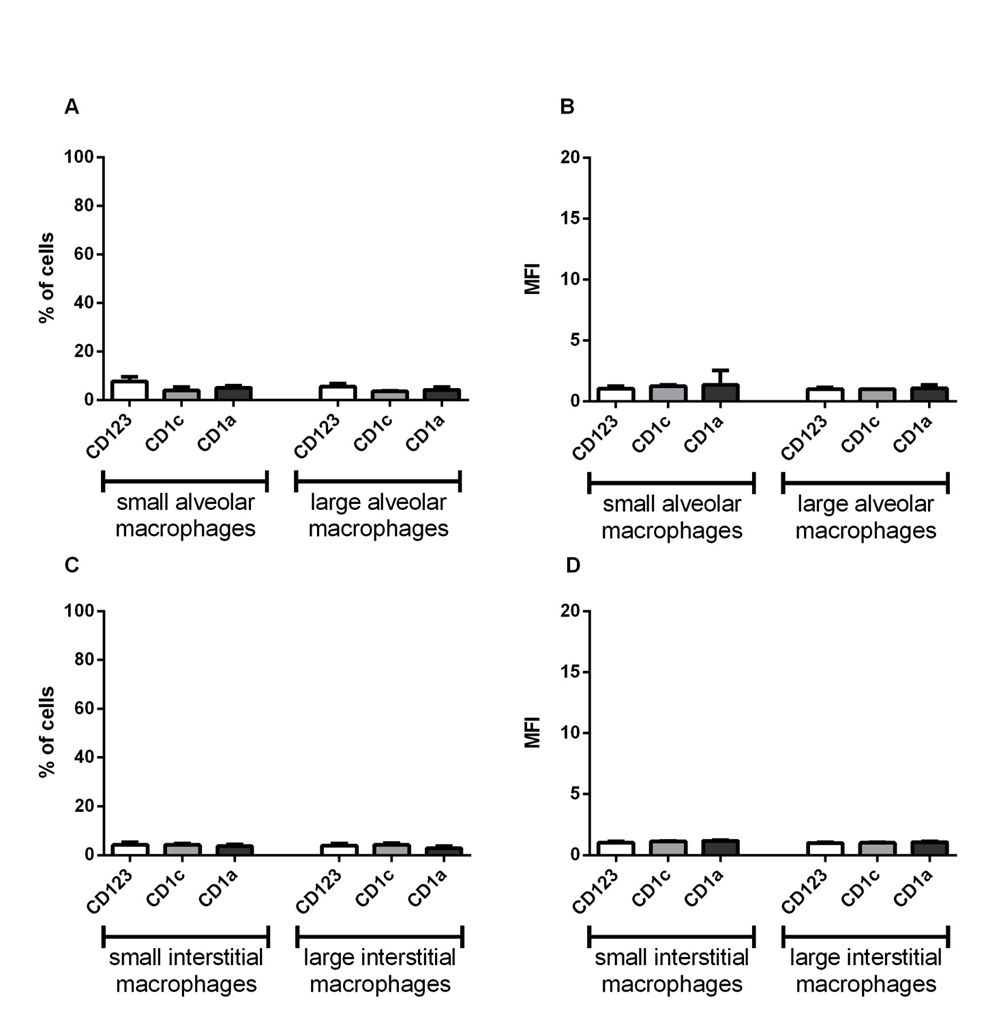


**Figure S5 The expression of dendritic cell markers in macrophage subpopulations.** Flow cytometric analysis of enriched alveolar (A and B) and interstitilal (C and D) macrophages for n=7 patients. Markers analysed were CD123, CD1c and CD1a. Data is expressed as the percentage of cells within each subpopulation expressing a specific marker (A and C) and the median fluorescence intensity (MFI) of each marker (B and D) and represents mean (SEM).

**
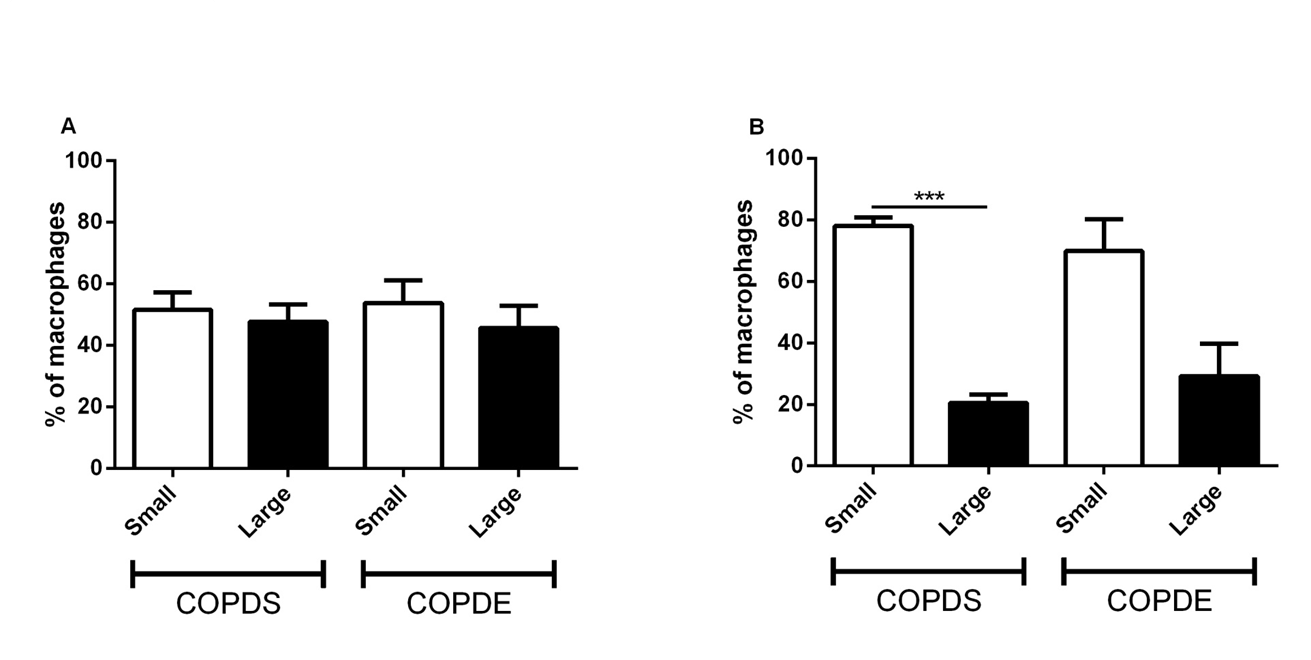
**

**Figure S6 The proportion of small and large alveolar and interstitial macrophages in COPD (including smoking status).**

Alveolar (A) and interstitial (B) macrophages were isolated by EasySep purification. A gate was drawn around the macrophages and the proportions of macrophage subpopulations were analysed within this gate by flow cytometry (Figure E3). The number of small and large macrophages present was expressed as a percentage of the total macrophage population. Data represents mean (SEM) of n=11 COPD smokers (COPDS) and n=7 COPD ex-smokers (COPDE) alveolar macrophages (A) and n=6 COPDS and n=5 COPDE interstitial macrophages (B). Paired t test (two tailed) was performed. ***= p<0.0001 significantly higher proportion of small interstitial macrophages compared to large interstitial macrophages.

**Figure S7 The expression of macrophage markers in macrophages subpopulations from ICS users compared to non ICS users.** Flow cytometric analysis of enriched alveolar small (A and B), large (C and D) and interstitial small (E and F) and large (G and H) macrophages for n=10 non ICS users and n=3 ICS users COPD patients. Markers analysed were HLA-DR, CD14, CD38, CD36, CD206 and CD163. Data is expressed as the percentage of cells within each subpopulation expressing a specific marker (A, C, E and G) and the median fluorescence intensity (MFI) of each marker (B, D, F and H) and represents mean (SEM). Paired t test (two tailed) was performed for each marker.

**Figure S8 Expression of MARCO RNA in macrophage subpopulations.** Alveolar (AM) and interstitial (IM) macrophage subpopulations were isolated using EasySep monocyte enrichment kit and FACS. Cells were lysed and RNAs were extracted and gene expression of MARCO was assessed by RT-PCR. Data represents mean (SEM) of n=8 COPD patients. Relative expression levels were determined using the ΔCt method normalizing to the house keeping gene (GAPDH). *** represent significant difference of MARCO expression between macrophage subpopulations (p<0.001).

**
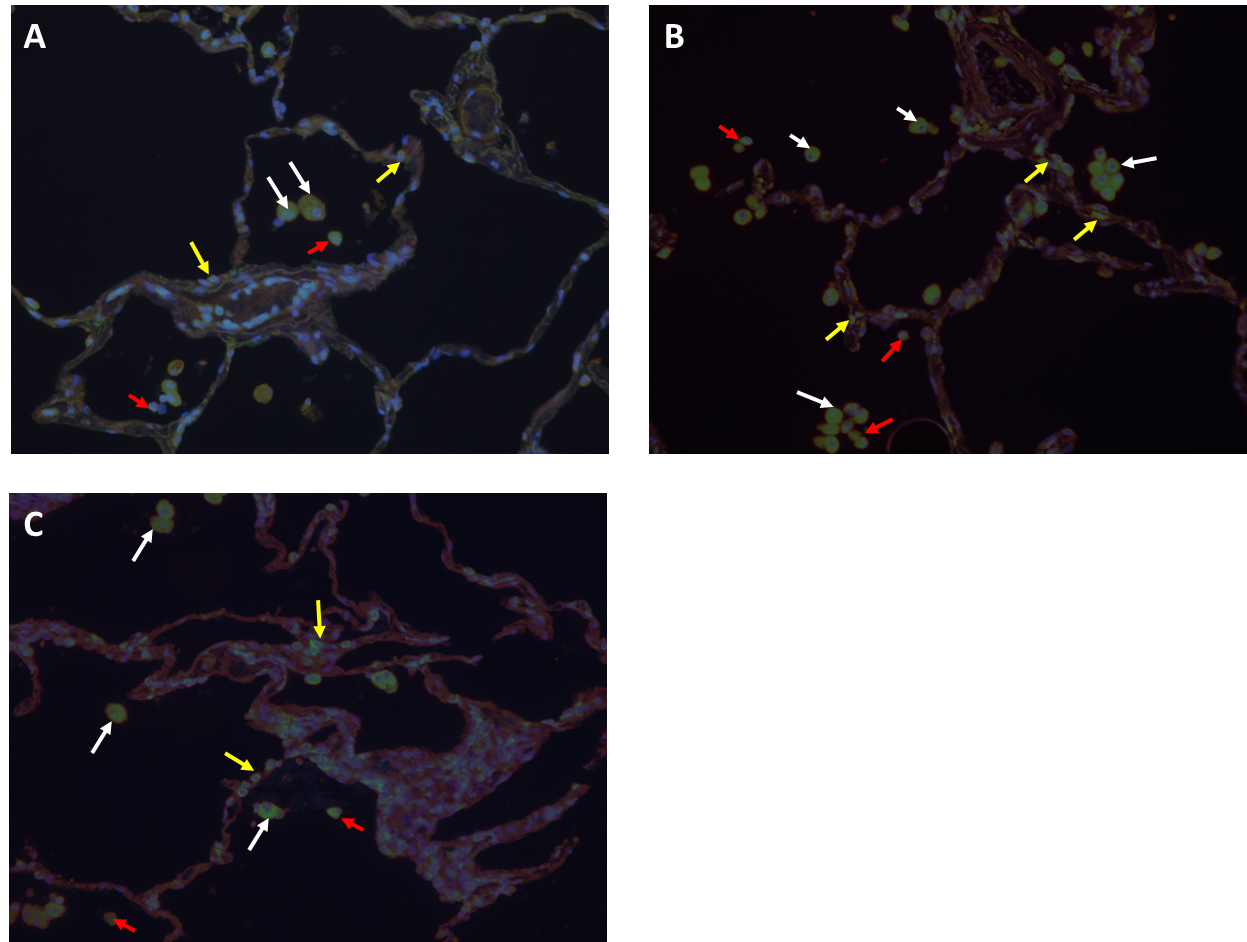
**

**D**

**Figure S9 Identification of alveolar and interstitial macrophages in lung resected tissue.** Alveolar (AM) and interstitial (IM) macrophages from COPD patients (n=12), S (n=15) and NS (n=11) were identified in FFPE resected lung tissue by co-expression of CX_3_CR1 and CD14 and the diameter of each cell was measured. Representative images are shown from COPD patients (A and D), smoking controls (B) and never smokers (C). Macrophages located in the alveolar spaces were defined as alveolar macrophages (AM) and showing a range of size with large cells (white arrows) and small cells (red arrows) and macrophages located in the alveolar walls or peripheral tissue were defined as interstitial macrophages (IM) (yellow arrows). D shows unstained alveolar macrophages from a current smoking COPD patient with or without carbon particles intracellularly (black and green arrows respectively).

**
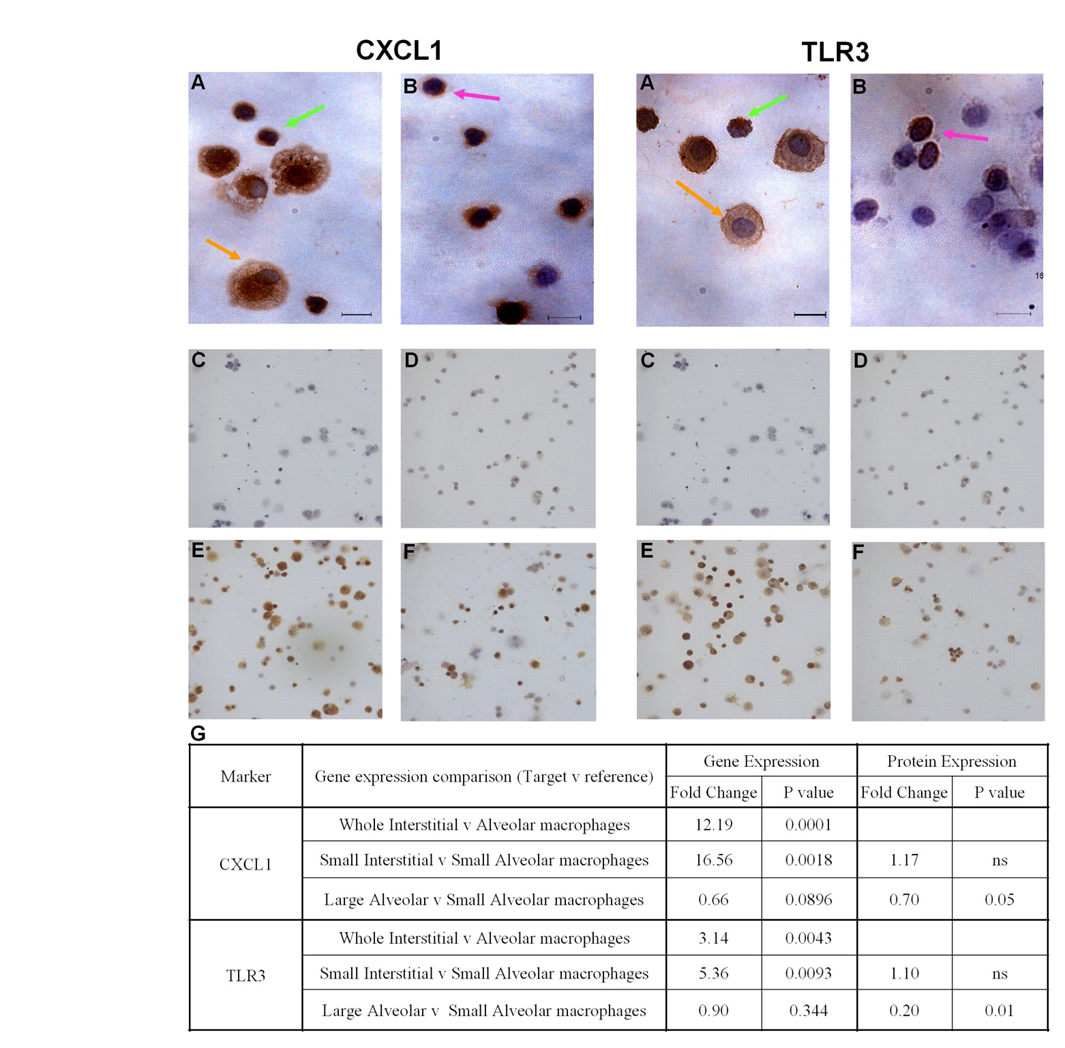
**

**Figure S10 Expression of CXCL1 and TLR3 in macrophage subpopulations.**

Alveolar and interstitial macrophages were isolated by EasySep purification. Representative images of alveolar and interstitial macrophages at x40 are shown in A and B respectively. Small alveolar (green arrow) and large alveolar (orange arrow) macrophages are shown in A. Small interstitial macrophages (pink arrow) are shown in C. Images of alveolar and interstitial macrophages at x20 are shown in C, E and D, F respectively. Negative controls are C and D. The scale bar represents 16 µms. A summary table of corresponding gene and protein expression is shown in G.

**
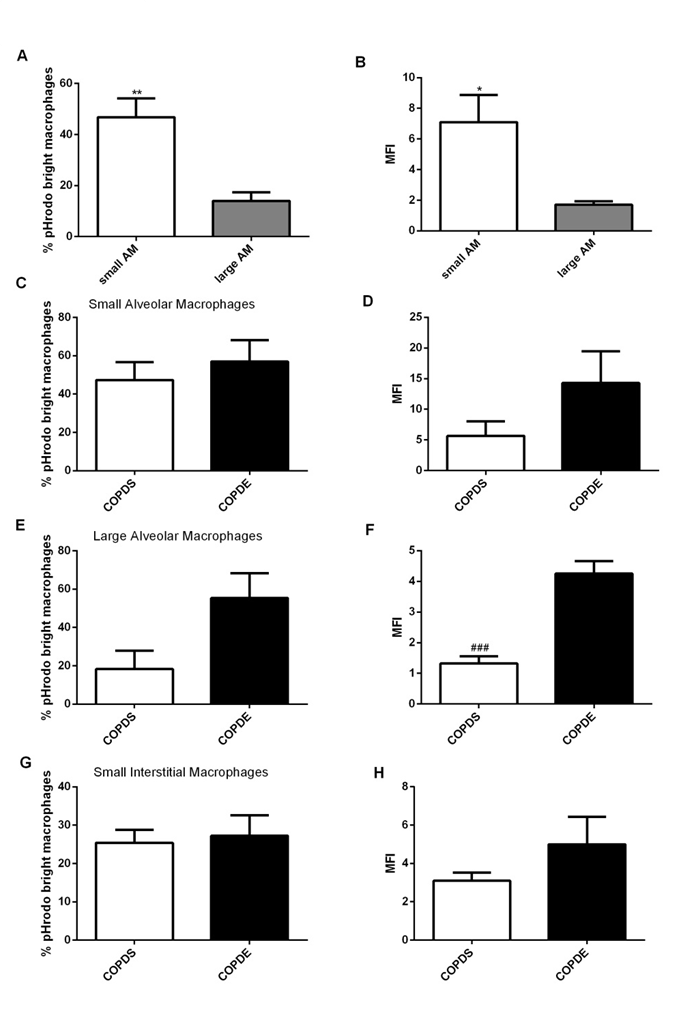
**

**Figure S11 Phagocytosis of pHrodo E. coli BioParticles by macrophage subpopulations from COPD patients (including smoking status) and alveolar macrophage subpopulations from Smokers without airway restriction.**

Alveolar and interstitial macrophages were isolated by EasySep purification. A gate was drawn around the macrophages and the ability of macrophage subpopulations to phagocytose pHrodo E. coli BioParticles in 1 hour was analysed by flow cytometry. The phagocytic ability of small alveolar macrophages was compared to large alveolar macrophages from smoking patients (S: n=7) (A and B). The phagocytic ability of small alveolar (C and D), large alveolar (E and F) and interstitial macrophages (G and H) from COPD smokers (COPDS: n=6) compared to COPD ex-smokers (COPDE: n=3) was analysed. Data is represented by % of pHrodo bright macrophages within each subpopulation (A, C, E and G) and median fluorescence intensity (MFI) of each subpopulation relative to the negative control (B, D, F and H). Data are shown as mean (SEM). Paired t test (two-tailed) was performed. *, **= significantly higher phagocytic ability of small alveolar macrophages compared to large alveolar macrophages (p<0.05, <0.01 respectively). ### = significantly lower phagocytic ability compared to COPDE (p<0.001)

**
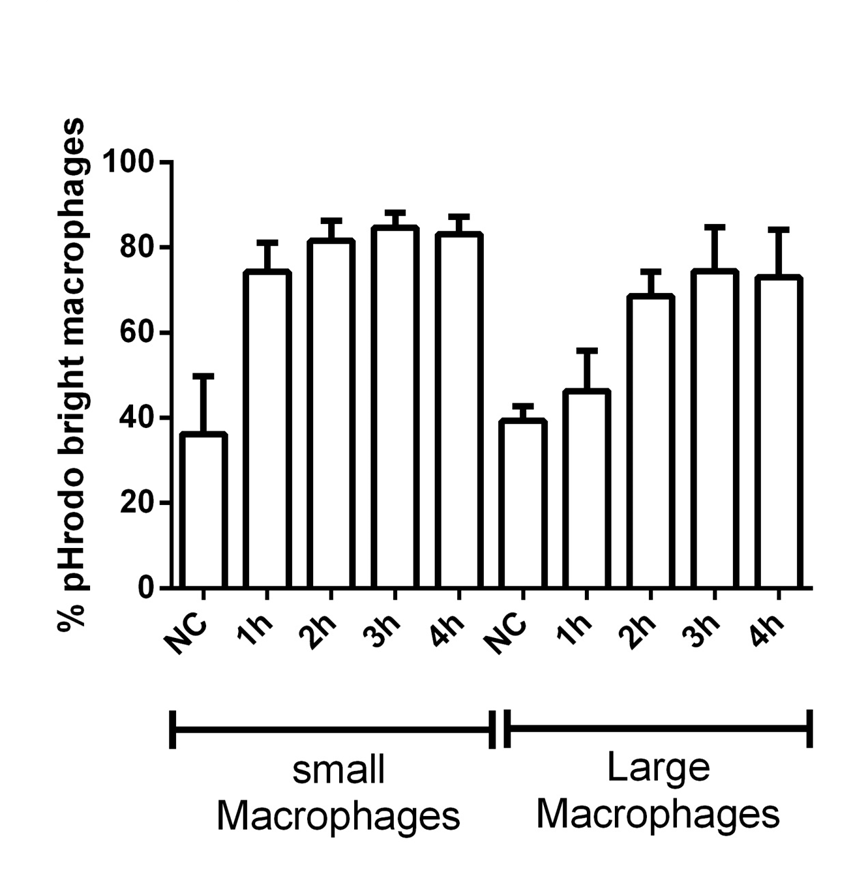
**

**Figure S12 The sub optimal time for phagocytosis of pHrodo *E. coli* BioParticles in small and large alveolar macrophages.**

Alveolar macrophages from COPD patients and smokers (n=3) were isolated using EasySep monocyte enrichment kit and phagocytosed pHrodo *E. coli* BioParticles for 1h, 2h, 3h and 4h in a shaking incubator. Negative control cells (NC) phagocytosed for 4 hours but were kept on ice. Phagocytosis was analysed by flow cytometry. Data are shown as mean (SEM).


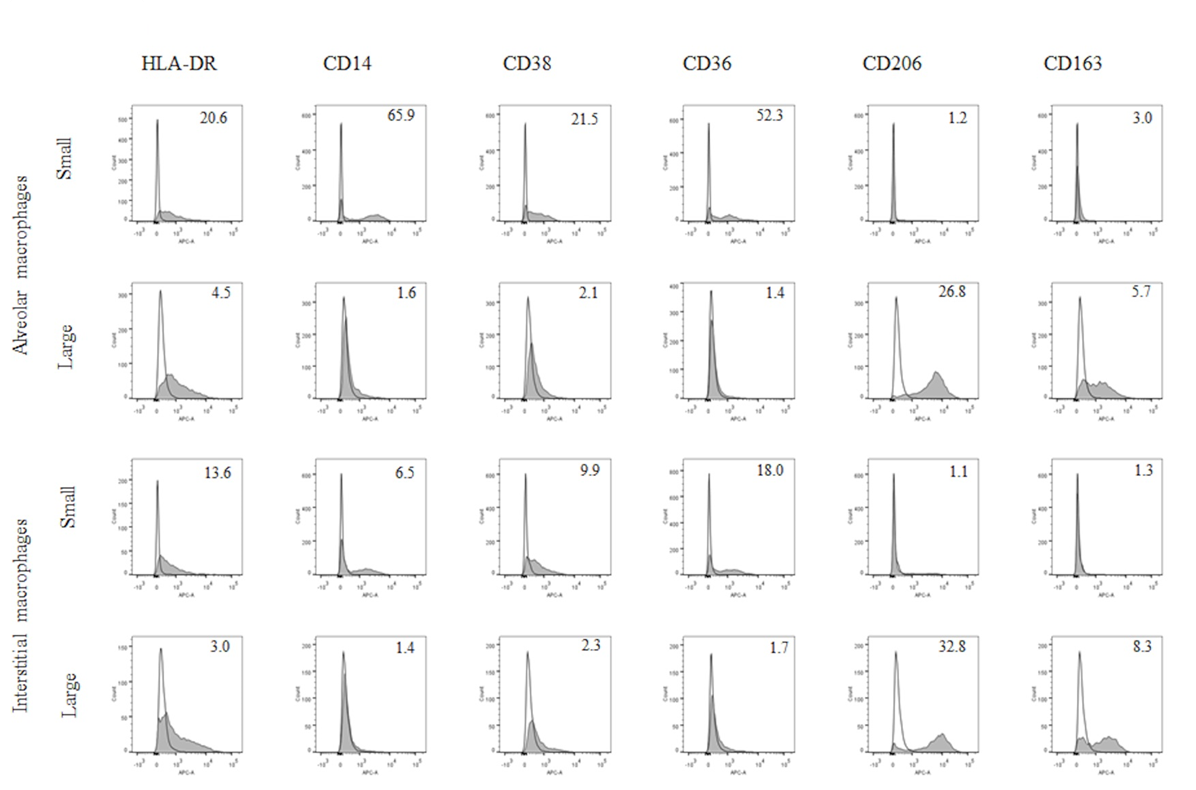
**Figure S13 Representative raw flow cytometry histograms of macrophage marker expression in small and large macrophages**.

Alveolar macrophage subpopulations are shown in the top panel and interstitial macrophages subpopulations are shown in the bottom panel. Isotype controls are shown as non-filled histograms. Markers analysed were HLA-DR, CD14, CD38, CD36, CD206 and CD163. The median fluorescence intensity (MFI) ratio was calculated and is shown on each histogram.

**Figure S14 Representative raw flow cytometry histograms of macrophage marker expression in large alveolar macrophages from COPD patients (including smoking status)**.

Isotype controls are shown as non-filled histograms. Markers analysed were HLA-DR, CD14, CD38, CD36, CD206 and CD163. The median fluorescence intensity (MFI) ratio was calculated and is shown on each histogram.

**
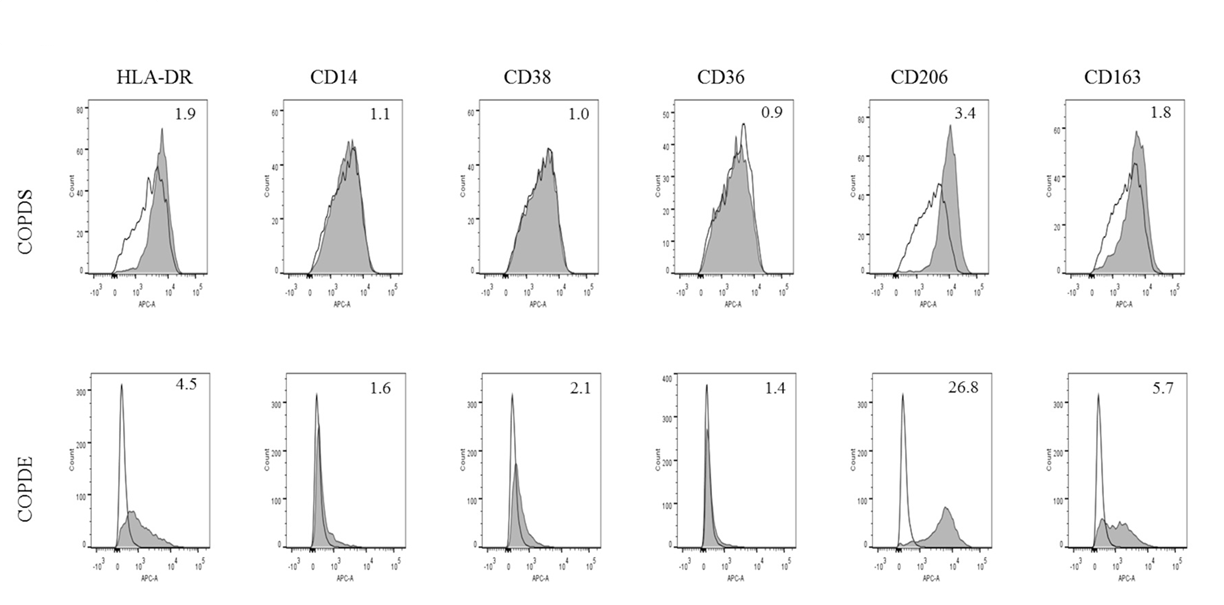
**
